# From localization to distribution: revisiting the functional organization of the extrastriate body area

**DOI:** 10.1093/cercor/bhag078

**Published:** 2026-07-09

**Authors:** Vojtěch Smekal, Lena L M Beckers, Marta Poyo Solanas, Beatrice de Gelder

**Affiliations:** Cognitive Neuroscience Department, Maastricht University, Oxfordlaan 55, Maastricht 6229EV, the Netherlands; Cognitive Neuroscience Department, Maastricht University, Oxfordlaan 55, Maastricht 6229EV, the Netherlands; Cognitive Neuroscience Department, Maastricht University, Oxfordlaan 55, Maastricht 6229EV, the Netherlands; Cognitive Neuroscience Department, Maastricht University, Oxfordlaan 55, Maastricht 6229EV, the Netherlands

**Keywords:** category selectivity, EBA, fMRI, network models

## Abstract

The extrastriate body area is a functional region of the human brain located in the lateral occipitotemporal cortex and was originally identified by its greater activation in response to images of human bodies compared with several other object categories. In subsequent research, the extrastriate body area was associated with a wide range of cognitive functions and processes localized to a substantial portion of the posterior cortex. In this review, we summarize prior research on the extrastriate body area and examine evidence linking the region to various cognitive processes. We also review the coordinates reported in the literature to better characterize its anatomical location. In addition, we report results from a 3-T functional MRI localizer for the extrastriate body area using highly controlled dynamic stimuli. Our findings indicate that extrastriate body area localization can be strongly influenced by the type of localizer stimuli used, the tasks engaging the region, and participant characteristics. Overall, the literature suggests that the role of the extrastriate body area extends beyond the visual representation of the human body to encompass broader cognitive and affective processes. This pleads in favor of orienting our understanding of the extrastriate body area toward the role of body perception in behavior.


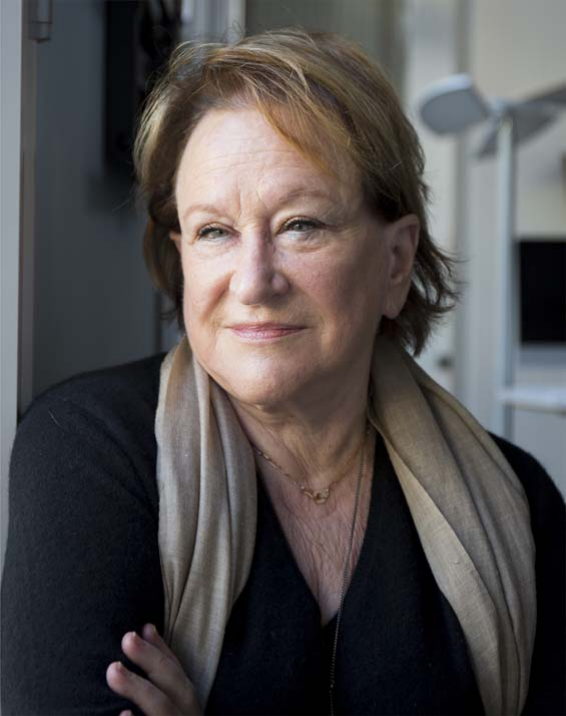


**Beatrice de Gelder** is Professor of Cognitive Neuroscience in the Faculty of Psychology and Neuroscience at Maastricht University in the Netherlands, and a member of the Maastricht Brain Imaging Centre (M-BIC). Previously she held positions in Leuven, Leiden and Tilburg University and at the Martinos Center for Biomedical Imaging, Harvard University. She received an MA in Philosophy, an MA in Experimental Psychology and a PhD in Philosophy from Leuven University in Belgium. Current research focuses on face and body perception including studies of residual functions in brain damage. She coordinated various EC grants in FP6 and FP7 and is a partner in two H2020 consortia. She was the recipient of an ERC grant in 2012 and co-recipient of an ERC Synergy grant in 2019. Extensive documentation at www.beatricedegelder.com

Bodies and body movements are important social signals, providing information about a person’s actions, feelings, and intentions. The importance of human bodies for navigating the visual and social environment has led to the proposal that specific brain regions are specialized for processing the visual appearance of bodies. This idea emerged from influential models of brain organization in cognitive science during the late 1980s, which emphasized the concept of cognitive modules (Bruce and Young 1986) and the existence of dedicated neural representations for specific visual categories. This led to identifying brain areas responsible specifically for processing different visual categories, for instance, faces (fusiform face area; Kanwisher et al. 1997) and places (parahippocampal place area; Epstein et al. 1999). Similarly, the extrastriate body area (EBA) was reported to be the area processing images of bodies (Downing et al. 2001).

Downing et al. (2001) conducted a functional magnetic resonance imaging (fMRI) study and found a region in the right lateral occipitotemporal cortex (LOTC) that exhibited a greater percent blood oxygen level–dependent signal change for images of human body parts and face parts compared to object parts. The authors further showed that responses to whole-body images were even stronger than those for body parts. The area showed a minimal response to images of whole faces, and the preference for body parts over object parts persisted even when stimuli were presented as line drawings rather than photographs. Following testing for potential confounding factors, the authors argued for the existence of a “specialized neural system for the visual perception of the human body.”

Some studies aimed to further elucidate the exact nature of the representation of the body in the EBA (eg Downing et al. 2006; Pitcher et al. 2019), while other whole-brain studies turned to body perception as part of action (Schubotz and von Cramon 2004; Kable and Chatterjee 2006) and emotion perception (de Gelder et al. 2004; Peelen et al. 2007). Efforts to understand how body-related representations in EBA contribute to broader cognitive processes, together with findings that EBA response to identical stimuli may vary depending on task demands (Marrazzo et al. 2021), have led to a shift in how this region is conceptualized. Proposals suggest moving away from the classical view of the EBA as purely a node for the visual representation of the body and instead moving to viewing it as a highly connected region involved in higher-order computational processing, with its functional properties modulated by task and context (de Lange and Bekkering 2011; Lingnau and Downing 2015). This aligns with a general move toward considering category representation in the brain in the context of behavior (Bracci and Op de Beeck 2023; de Gelder and Solanas 2021; Li et al. 2025; Ritchie et al. 2025).

Advancing a behavior-oriented understanding of the EBA requires a comprehensive overview of the functions in which the region is involved and its role within these processes. Developing a framework that conceptualizes the EBA as a multimodal and highly interconnected region therefore requires integrating the diverse findings on the area into a unified account. However, to our knowledge, a systematic review of the EBA literature is still lacking. Such a review would help clarify the range of functions associated with the region and provide support for a behaviorally grounded, context-dependent view of body representation. Addressing this gap is the first goal of the present paper.

In addition to the diverse findings on EBA function, the literature also shows considerable variability in the reported anatomical location and properties of the region. This variability in reported anatomical location can be caused by differences in stimuli and analysis approaches used to localize the region, as well as the task and context modulation. Thus, the field would benefit from a thorough examination of the reported locations of the EBA, and the methods used for localizing the region, as well as a carefully conducted and replicable localization of the region to serve as future reference. This is especially relevant because the region has increasingly been targeted in noninvasive brain stimulation (NIBS) studies, which commonly depend on coordinates reported in prior research to guide stimulation.

In sum, this contribution pursues three main objectives: to examine the functional role of the EBA, clarify its anatomical location, and consider its broader relevance for behavior. First, we systematically map the functional profile of the EBA, highlighting the relevance of a shift in perspective and providing a structured overview to guide future research on the region (Part I). Second, we review the various methods used to localize the EBA, evaluate how methodological differences may have shaped reported findings, and synthesize a more comprehensive account of its anatomical location (Part II). Finally, we compare the heterogeneous anatomical results reported in the literature with findings from a carefully controlled EBA localizer (Part III). Together, these analyses aim to provide an integrated reference framework for future investigations into the functional and anatomical properties of the EBA.

## Part I—Literature review

### Methods

**Figure 1:**
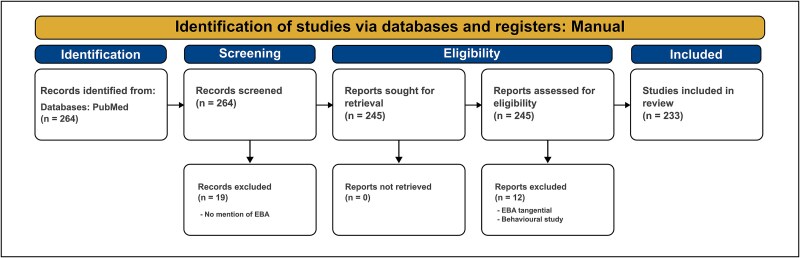
Literature review PRISMA diagram. Diagram explaining the procedure for article selection in the literature review as well as the coordinate review.

The initial literature search was conducted using PubMed and the search term “extrastriate body area” on 2024 March 18. This resulted in the identification of 264 articles. The relevance of each article was assessed by author V.S. following the procedure depicted in Fig. 1. First, article abstracts were assessed and any which did not relate to psychology or neuroscience and the EBA were excluded (*n* = 19). Then, the remaining articles were screened and any articles which discussed the EBA only tangentially and the findings of which did not relate to the EBA were also excluded (*n* = 12). These articles were then grouped based on their research focus and, for empirical studies, by the methodology used (see Supplementary Table S1 for a summary of articles by research focus). For each article, reported EBA coordinates as well as the stimuli used to identify the EBA were extracted whenever available (see Part II).

## Results

**Figure 2:**
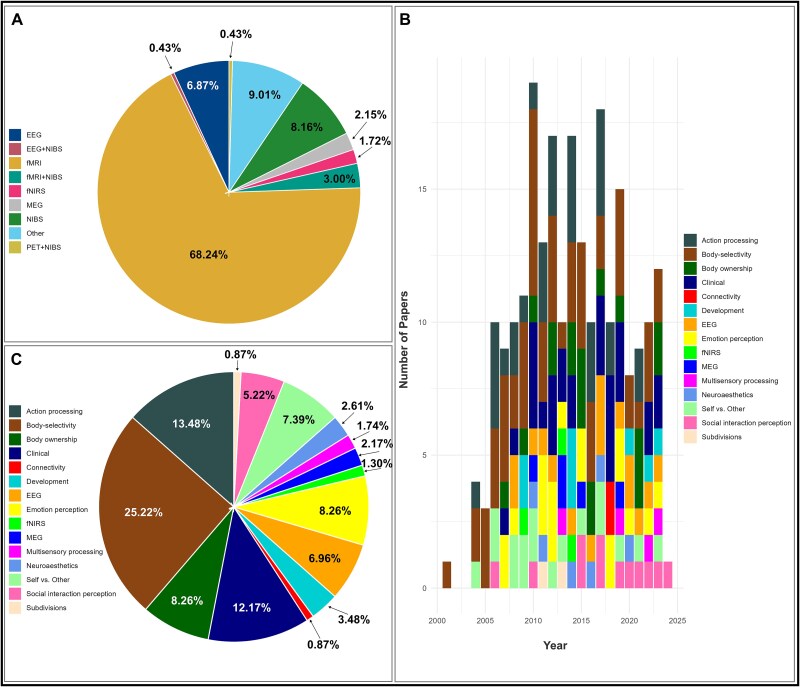
Summary of EBA literature review findings. (A) Pie chart showing the different methodologies used in the studies included in the literature review (Part I). “Other”: Commentary papers, reviews, meta-analyses, intracranial recording studies, lesion-mapping, behavioral research, opinion papers, and cytoarchitectonics research. (B) Histogram of the articles included in the literature review showing the number of articles published in a given year and divided by research topic. (C) Pie chart showing the distribution of research topics among the collected papers. Based on data collected on 2024 March 18. Abbreviations: EEG, electroencephalography; TMS, transcranial magnetic stimulation; fMRI, functional magnetic resonance imaging; NIBS, noninvasive brain stimulation (includes TMS, transcranial direct current stimulation [tDCS], and transcranial alternating current stimulation [tACS]); fNIRS, functional near-infrared spectroscopy; MEG, magnetoencephalography; PET, positron emission tomography.

Most of the articles included in the final literature review consisted of fMRI studies (68.24%). An overview of the frequency of methods used can be seen in Fig. 2A. As Fig. 2B shows, there has been a relatively consistent publication rate of articles concerning the EBA since around the year 2007 up to the present day. The collected articles were also divided by research topic, which resulted in a total of 15 research topic categories and a relatively broad distribution of the articles among the topics (see Fig. 2C and Supplementary Table S1). Each research topic and the articles included within are now summarized in turn.

### Body selectivity

The EBA was first identified as a body-selective area, showing greater activity for visual presentation of human bodies compared to other images. Since then, research has explored the boundaries and flexibility of this selectivity, examining responses to body parts, comparing dynamic and static stimuli, and even extending beyond vision to non-visual presentation of bodies using tactile body perception (Constantini et al. 2011).

#### Whole-body selectivity

The selectivity of the EBA for human body stimuli has been thoroughly examined by comparing its responses to a wide range of control object categories. Across many studies, the strongest activity has consistently emerged for bodies. For example, Downing et al. (2006) compared 20 different image categories and found the strongest responses for bodies. Other work showed stronger EBA responses for bodies than for face-only or face-and-body stimuli (Morris et al. 2006), for bodies versus places and a computer character looking at occluded bodies and places (Heyda et al. 2010), and for bodies relative to objects, faces, mammals, birds, fruits, and sculptures (Caspari et al. 2014), or robot/android movements (Saygin 2012). While initial work by Downing et al. (2007) suggested overlapping activation for objects, bodies, body parts, and motion stimuli, follow-up region-of-interest (ROI)-based and multivoxel analyses showed non-overlapping, independent patterns of activity for each stimulus type. Murty (2021) developed an artificial neural network–based encoding model for the EBA, which showed distinctly human body–like stimuli as preferentially activating the EBA. Other studies extended these results, showing that even stylized or socially meaningful depictions of bodies drive EBA responses, such as for example drawings of mourning body postures from various cultures (Labek et al. 2017) or features such as body sex and weight (Foster et al. 2019).

While such studies provided support for the EBA’s role in visual body perception, transcranial magnetic stimulation (TMS) studies were able to provide causal evidence for the region’s involvement in this function (see Downing and Peelen 2016 for a previous review of TMS studies on the EBA). For example, Urgesi et al. (2004) were the first to demonstrate this by applying repeated transcranial magnetic stimulation (rTMS) over the EBA during a visual discrimination task for bodies, faces, and non-body objects. EBA stimulation, and not V1 stimulation, led to a significant increase in reaction time for body stimuli only. Pitcher et al. (2009) found matching results but used the occipital face area and lateral occipital area (LO) as their control stimulation sites. van Koningsbruggen et al. (2013) showed that right EBA stimulation impaired people detection but not car detection in both visual hemifields. Gandolfo and Downing (2019) also showed that EBA stimulation eliminated a cueing facilitation effect of body stimuli and not scene stimuli, while occipital place area stimulation had the opposite effect.

Research has also addressed the timing and dynamics of EBA responses during body perception. For example, Pitcher et al. (2012) found two critical time periods (40 to 50 ms and 100 to 110 ms after stimulus onset) in which applying TMS to the EBA impaired performance on a body-perception task. Pourtois et al. (2007) also presented strong evidence for the EBA’s body-selectivity when the authors recorded intracranial local field potentials from an epilepsy patient while viewing bodies, faces, mammals, and tools. A body-selective response restricted to the LOTC with activity starting from 190 ms and peaking 260 ms after stimulus onset was found. Additionally, Kim et al. (2020) found spontaneously evoked patterns of brain activity in the EBA to be related to body-evoked patterns, suggesting a representational function for spontaneous activity.

Finally, lesion studies have provided further evidence for the EBA’s body-selectivity. Moro et al. (2008) found that lesions in the EBA impaired recognition of bodies but not faces and objects. These lesions specifically disrupted body form recognition without affecting body action recognition. Van den Stock et al. (2014) presented images of bodies, faces, butterflies, cars, and their scrambled counterparts to a patient with a complete V1 lesion and still found significant EBA activation for body stimuli.

#### Body-part selectivity

With the selectivity of the EBA for images of the body firmly established, the question arose whether the EBA also responded to images of body parts. Indeed, various studies seem to support this notion. For instance, Spiridon et al. (2006) found more consistent EBA activation for body parts than objects. Cziraki et al. (2010) found adaptation effects for hand images both behaviorally as well as neurally, as indicated by a reduction in EBA activity. Fusco et al. (2022) applied transcranial alternating current stimulation (tACS) to the medial frontal cortex and EBA and found improved performance on a hand-flanker task compared to a task with images of letters. In another study, Andres et al. 2012 found significant EBA activity during a finger discrimination task, further supporting the EBA’s involvement in processing body parts.

However, not all findings align with this body-part specificity. Bracci et al. 2010 identified a region responding to images of hands, fingers, and feet, and this region was partially overlapping but distinct from the EBA. Although the EBA did show significant activity for images of arms, legs, torsos, hands, and feet, it did not respond to images of fingers. This suggests a nuanced role of the EBA in body-part processing, one that may be less sensitive to smaller body parts. Supporting this view, Perruchoud et al. 2016 found greater EBA activity for mental rotation of the whole body compared to mental rotation of the hands.

Further studies have explored the relationship between whole-body and body-part processing in the EBA. For instance, Taylor et al. 2007 found EBA activity to gradually increase with increasing visibility of the body and Brandman and Yovel 2016 showed greater EBA activity for whole bodies compared to configurations of individual parts. Blanke et al. 2010 also found higher left EBA activity for mental imagery of whole bodies compared to partial bodies. Urgesi et al. 2007 applied TMS to the right EBA, which led to impaired performance on a match-to-sample task for inverted bodies only and not upright bodies, suggesting local body processing compared to configural processing in the right EBA. Only Bauser and Suchan 2015 did not find significant differences in EBA activation between whole bodies and bodies scrambled at the body-part level.

Overall, the research suggests that the EBA is selective not just for bodies but also for body parts, although the selectivity for whole bodies is greater than for smaller body parts and greater for large body parts, such as limbs, than for smaller parts, such as fingers.

### Dynamic stimuli

The EBA has also been shown to respond more strongly to dynamic body stimuli than to static ones. Felician et al. (2009) reported greater EBA activity when participants viewed videos of bodies compared to photographs or drawings. Similarly, Pitcher et al. (2019) observed stronger EBA responses to moving bodies than to static images. One possible explanation for this effect was proposed by Stigliani et al. (2015), who demonstrated that the EBA has a significantly higher temporal processing capacity—meaning it can process more information per unit of time—than other category-selective visual regions.

There have since been several contradictory findings about the contribution of dynamics to EBA activity. Michels et al. 2005 found EBA activation for biological motion stimuli without local image motion; however, the results of Vangeneugden et al. 2014 suggested that the EBA processed body form and not body motion. Similarly, Gilaie-Dotan et al. 2015 found that an EBA lesion did not impair biological motion perception from point-light displays. Poyo Solanas (2020a, 2020b) correlated brain activity with computation-based body features and found EBA activity to be more in line with postural rather than kinematic features of the body. Similarly, Marrazzo et al. 2023 found the EBA to represent the body by a combination of low-level visual and postural features. Efforts have been made to reconcile these conflicting views, and Thompson and Baccus 2012 found independent contributions of body form and body motion to EBA activity, with Duarte et al. 2022 demonstrating a similar finding.

Some of these seemingly contradictory findings may be explained by the action-related region (ARR) identified by Peelen and Downing (2005). In replicating the fMRI study by Astafiev et al. (2004), which showed that arm and foot movements toward a visual target elicited EBA activity even without visual feedback, Peelen and Downing demonstrated that the region responding to body-part movements was not identical to the EBA. Instead, they identified a distinct but partially overlapping region, termed the ARR. When localized using a functional localizer, the ARR overlapped with the EBA by only 19%, suggesting that previous reports of EBA involvement in action execution may partly reflect activity in this neighboring region rather than the EBA itself.

### Viewpoint invariance

Next to this, in a series of fMRI experiments, Taylor et al. 2009 demonstrated that body representation in the EBA is view invariant. EBA activity adapted to identical views of the same pose with the degree of adaptation varying with the size of the viewpoint difference. Similarly, Ewbank et al. 2011 found EBA activity attenuated by repeated presentation of bodies with size- and view-invariant representations. On the other hand, Foster et al. 2022 found that the EBA encoded stimulus orientation for both faces and bodies, independent of whether attention was on the stimulus orientation or identity.

### Non-visual body selectivity

The EBA is not limited to supporting visual perception but also shows sensitivity to body-related information from other sensory modalities. For example, Kitada et al. 2009 found that the EBA responded to both visual and haptic presentation of hands and feet during a recognition task. Similarly, Constantini et al. 2011 found bilateral activity in the EBA for haptically presented body parts compared to objects.

In addition to sensory processing, the EBA may also be involved in conceptual and linguistic representations of the body. Kemmerer and Tranel 2008 investigated lexical and conceptual knowledge about body parts in 104 brain-damaged patients. Eight patients were impaired on naming of body parts, including one individual with a lesion covering the left EBA. Rueschemeyer et al. 2010 showed that words denoting objects typically brought toward the body activated the EBA. Lacey et al. 2017 found EBA activity for metaphorical sentences involving references to body parts, but no such activation for literal sentences referring to them. In line with this, resting-state fMRI analysis showed connections between the EBA and ipsilateral semantic processing regions.

Together, these findings suggest that the EBA is a multisensory and multifunctional region, extending beyond visual perception to support body-related processing across sensory, conceptual, and linguistic domains.

#### Demographic differences

Aleong and Paus 2010 showed that activity in the right EBA was higher for women than for men when viewing images of human bodies. Women also showed higher activity in the right EBA compared to the left EBA. The authors related these differences to sex differences in perception and evaluation of one’s own body. Additionally, Willems et al. 2010 found that the EBA was right lateralized in right-handed people, but not in left-handers.

#### Body processing beyond a categorical EBA

Body perception has also been related to other areas in the brain, which have shown significant activation for body stimuli. Taylor and Downing 2011 conducted a review of studies comparing category-selective areas in the posterior-lateral occipitotemporal cortex with a particular focus on the division of labor between pairs of category-selective areas, including the EBA and fusiform body area (FBA). EBA representations were more primitive, local, and stimulus driven compared to the FBA representations. Li et al. 2023 combined ultrahigh-field fMRI with independent component analysis to show that the EBA is part of a large-scale network involved in human body processing, which also includes the superior temporal sulcus (STS), temporoparietal junction (TPJ), premotor cortex, and inferior frontal gyrus (IFG). Corradi-Dell’Acqua et al. 2015 showed greater EBA activity when attention was drawn to bodies rather than to buildings, but only when the saliency of the input was reduced using background noise, suggesting attentional top-down modulation during weak bottom-up signals. Additionally, Zeharia et al. 2019 had subjects move 20 different body parts during fMRI and found evidence of a body-part topography in the precuneus, with each body-part region showing connectivity with the EBA. Together, these studies illustrate that the EBA is a highly connected region and that body processing is not an isolated process within the EBA but involves an extended neural network.

### Connectivity

As discussed in the previous sections, the EBA does not function in isolation; rather, it operates as part of a broader network, dynamically interacting with multiple brain regions to support the diverse body-related processes in which it is involved. An important investigation into EBA connectivity was conducted by Zimmermann et al. 2018, who used resting-state fMRI and diffusion-weighted MRI to examine its connectivity with the dorsal and ventral visual pathways. The authors found the EBA to be more strongly connected to dorsal regions compared to regions of the ventral stream, such as the FBA or the LOC. This pattern of connectivity was interpreted as evidence for the EBA’s involvement in goal-directed action planning.

Various other studies included throughout this review also report findings on EBA connectivity. For example, Zeharia et al. 2019 found connectivity between the EBA and body part-selective regions in the precuneus. Several studies identified functional coupling between the EBA and the parietal cortex during visuoproprioceptive paradigms (eg Limanowski and Blankenburg 2015; Limanowski and Blankenburg 2017; Moayedi et al. 2021). Tomasino et al. 2012 showed increased coupling between the EBA and a personal space network when arm representation was modified by imagining tool use. Hamamoto et al. 2023 found EBA and anterior insula connectivity to be correlated with body-size overestimation. Jung et al. 2020 showed modulation of the connectivity between the EBA and a fronto-insular-temporal network when participants imagined fear-associated and disgust-associated bodily sensations. Both Van den Stock et al. 2015 and Bellot et al. 2021 found greater coupling between the EBA and the STS during social interaction perception, while Greven and Ramsey 2017 showed stronger coupling between the EBA and the theory-of-mind network when participants viewed familiar individuals versus strangers. Okamoto et al. 2020 identified stronger coupling between the EBA and the IFG when participants initiated actions themselves rather than simply observing others. Finally, both Ma et al. 2018 and Gu et al. 2021 showed modulated connectivity between the EBA and a wide range of other regions (including hMT+ and the pSTS) during action perception.

Interestingly, in a different line of research, Rebollo et al. 2018 identified a so-called gastric network, based on connectivity of the brain with an electrical rhythm generated by the stomach. A key feature of this network was a strict temporal alignment between the gastric cycle and various brain regions. In the final stages of this temporal synchronization, the EBA emerged as a key region exhibiting tight temporal alignment with the gastric cycle.

Other connectivity results related to the EBA were undertaken in the context of clinical disorders. These results are discussed in the respective sections.

### Self-versus-other discrimination

An interesting issue is whether the EBA distinguishes between self and others. Research investigating these questions has taken two main approaches (see Schurz et al. 2013 and Arora et al. 2017 for previous reviews on EBA involvement in perspective taking). One line of research has compared EBA responses to different perspectives—from an actor’s point of view (egocentric) or from an observer’s point of view (allocentric). The other line of research has directly compared stimuli that represent the participant’s body versus stimuli of others’ bodies.

Findings on the first question have been contradictory. Chan et al. 2004 found significantly higher right EBA activity for allocentric compared to egocentric views of both the participants’ own and others’ bodies. Saxe (2006) replicated these findings and Cazzato et al. 2015 found that rTMS over the EBA impaired performance on a body-size judgment task for allocentric perspectives only. Callan et al. 2012 found higher EBA activity for the allocentric perspective, even in the absence of the body as a reference point. On the other hand, Jackson et al. 2006 found no differences in EBA activity for egocentric and allocentric perspectives and De Bellis et al. 2017 showed that right EBA rTMS had no differing effects on visual perspective, while left EBA stimulation enhanced processing for the egocentric perspective. Finally, Carey et al. 2019 showed greater EBA activity for the egocentric perspective.

Studies comparing responses to images of one’s own body and others’ bodies also reported inconsistent results. Myers and Sowden 2008 found different parts of the EBA to be selective for either images of one’s own or others’ body parts. Vocks et al. 2010a meanwhile found greater EBA activity for self, compared to other images. Applying rTMS to the EBA, David et al. 2009 observed impaired performance for other-generated movement detection, and Pann et al. 2021 found TMS over the right EBA to induce more errors for others’ hands identification.

Finally, there are also studies suggesting that the EBA is not involved in the self–other distinction. Hodzic et al. 2008 found a region near but separate from the right EBA to be involved in this processing and this was replicated by Ogawa and Matsuyama 2023. Hodzic et al. 2009 found the EBA to be equally active for images of own and others’ bodies and Dumontheil et al. 2010 also showed that the EBA responded to images of bodies but was not involved in perspective taking.

The literature summarized in this section clearly shows that further work is needed to fully understand the role of the EBA in self-versus-other discrimination. Carefully constructed and controlled experimental paradigms taking the existing work into account may be necessary to establish the region’s contribution to this process.

### Body ownership/representation

Whether the region also differentiates between the self and others continues to be debated. A related question is whether the EBA plays a role in representing aspects of one’s own body, whether through proprioception or through subjective feelings of body ownership (see Di Vita et al. 2016, Seghezzi et al. 2019, and Bao et al. 2021 for meta-analyses and reviews on brain regions implicated in body representation and body ownership, including the EBA).

Many of the studies investigating this topic have made use of the rubber hand illusion (RHI) or a variant of the task (see Golaszewski et al. 2021 for a previous RHI study review). Wold et al. 2014 showed that rTMS over the left EBA could enhance the RHI leading to more misjudgments about real hand location, and Limanowski et al. 2014 found left EBA activity to correlate significantly with participants’ illusion scores. Limanowski Blankenburg et al. 2015 then induced self-attribution of an entire fake arm, which increased activity in the left EBA and functional connectivity between the EBA and the intraparietal sulcus. Limanowski Blankenburg et al. 2017 also found this increased connectivity for virtual hands. Finally, Moayedi et al. 2021 found EBA volume and its functional connectivity with the parietal cortex to relate to participants’ susceptibility to a finger-elongation illusion.

Another popular paradigm in this line of research is to have participants perform movements and then present either congruent or incongruent visual feedback of those movements, thereby inducing a match or mismatch between action and perception. However, this approach has yielded conflicting results when it comes to the EBA. David et al. 2007 found higher EBA activity for incongruent visual feedback during joystick movements. On the other hand, Kontaris et al. 2009 found no difference in EBA activity for matching or non-matching visual feedback for hand movements and Limanowski and Blankenburg 2016 found greater EBA activity for congruent visual arm position compared to incongruent visual information. Trying to resolve this, Sonobe et al. 2023 used multivoxel pattern analysis (MVPA) to decode congruent or incongruent visual feedback during tactile stimulation or motor execution of finger movements and found above-chance accuracy in the left EBA. Similarly, Beck et al. 2015 found that TMS over the EBA disrupted the enhancement of visual touch for the observation of either one’s own hand or another’s hand.

In addition, Tomasino et al. 2012 found that EBA activity was modulated by imagining using tools such as pliers, thereby changing the representation of the arm. Imagined tool use also increased functional connectivity between the left EBA and a network processing personal space around the hand (including the middle temporal gyrus, inferior frontal gyrus, and left parietal cortex). Moreover, Hummel et al. (2013) found evidence for neural adaptation to body shape manipulations in the FBA, but not in the EBA. However, Hamamoto et al. 2023 had participants estimate the width of their actual and ideal bodies and found connectivity between left EBA and anterior insula to positively correlate with overestimation of body size and connectivity of the left EBA with the right precuneus to negatively correlate with body dissatisfaction scores. Araujo et al. 2015 found higher EBA activity for questions to participants about their core self (interoception and exteroception) compared to questions about their autobiographical self (personality traits and biographical information).

Taken together, these findings suggest that beyond its well-known role in visually processing bodies, the EBA may also play a role in dynamic integration of visual, proprioceptive, and affective signals that sustain own body experience.

### Emotion perception

The first studies using fMRI to understand emotion perception from whole bodies contrasted neutral actions, angry (aggressive actions), and happy (welcoming actions) and looked at the whole brain (de Gelder et al. 2004). EBA was not localized separately as the focus was on differential emotion representation. Differential higher visual areas activity was found in areas covering LOC (lateral occipital cortex) as well as in the fusiform gyrus. This clearly supports the role of EBA in processing emotional body expressions but also puts this activity in the context of processes in other brain areas. This perspective has been borne out by later studies. Two studies specifically looked at EBA only came to opposite conclusions. Peelen et al. 2007 found that emotional body expressions resulted in significantly greater EBA activity than neutral bodies and that the EBA responses were positively correlated with amygdala activity. On the other hand, van de Riet et al. (2009) found the EBA did not significantly differentiate between different emotions or neutral bodies. This difference may have been caused by, for instance, stimuli differences (dynamic vs. static emotional expressions). A growing body of research since then, however, strongly argues for a role of the EBA in emotion perception (de Gelder 2016), but many variables that may play a role reflects the importance of bodily expressions of emotions in real life. Different studies have their own take on how to isolate the variables of interest. This often complicates comparisons across studies.

One collection of studies established the region’s role specifically in the perception of threat. Using dynamic stimuli, Kret and colleagues investigated brain regions involved in the perception of threat conveyed by the face or the body and found greater EBA activity for threatening bodies (Kret et al. 2011a). In male participants, the EBA also showed higher activity for bodily threat expressions depicted by male actors than female actors (Kret et al. 2011b). EBA activation for threatening body expressions was also correlated with participants’ levels of social inhibition (Kret et al. 2011c). Sinke et al. 2012 found that presenting threatening scenes can elicit right EBA activity even in the absence of an actual body present in the scene. The authors argued that the threatening scene gave rise to associative and anticipatory effects in the brain by activating areas related to bodily behavior associated with the scenes. Van den Stock et al. 2012 replicated many of these findings when investigating the perception of fear presented by bodies and scenes. Greater activity for fearful bodies compared to neutral ones was found in bilateral EBA. Additionally, a neutral body paired with a threatening background scene elicited a higher response in the EBA than with a neutral scene. This suggests an influence of emotional context information on body expression processing. Van den Stock et al. 2013 again found sensitivity for the affective content of a background scene in the EBA.

Other studies have focused on pain and on a variety of other emotions. Vachon-Presseau et al. 2012 found that images displaying pain-inducing agents to the hand or foot, or facial expressions of pain resulted in greater activity in the bilateral EBA compared to their neutral counterparts. Goldberg et al. 2015 compared emotion perception from different walking styles and showed greater activity within the EBA for emotional gaits compared to speed-matched neutral gaits. Yang et al. 2018 used MVPA to investigate EBA responses to a variety of face, body, and whole-person emotional stimuli. The authors found that whole-person patterns of activity were highly correlated with weighted sums of separate face and body patterns with different weights for happy stimuli compared to angry and fearful ones. These results thus argued for a body-part-based representation within the EBA, modulated by emotions. Caillaud et al. (2020) found that the EBA was more active when participants had to decode self-conscious emotions (eg pride, embarrassment) than other “basic” emotions (eg anger, surprise), suggesting the importance of body information for the self-conscious emotions. Ren et al. 2022 found that EBA responded more strongly to images of torsos and arms expressing anger and fear compared to a neutral emotion. Representational similarity analysis showed that the EBA activity allowed to distinguish between the different bodily expressions. Vaessen et al. 2023 found activity within the EBA for emotion expressions conveyed through the body and not through the face or voice.

Again, here there have been negative reports about EBA involvement in pain as well as in some emotion perception cases. Lamm and Decety 2008 investigated the involvement of the EBA in viewing pain perception in others contrary to the later findings of Vachon-Presseau et al. 2012. Participants were presented images of hands either in pain or not in pain, and all images of hands consistently activated the EBA with no difference in activity between the two conditions. Engelen et al. 2015 applied TMS to the IPL, EBA, and early visual cortex while participants were asked to identify fearful and neutral body expressions. While stimulation of the IPL had a significant effect specifically on fearful body recognition, EBA stimulation did not reveal any differences between emotion conditions. Van de Vliet et al. 2018 investigated emotional body perception in patients with anterior temporal lobe resections, which included the amygdala. The patients presented unimpaired performance on emotion categorization of body expressions and no differences in EBA responses compared to control subjects.

Other research has also suggested that not only the perception of emotions in others affects EBA activity but also the perception of emotions within oneself. Jung et al. 2020 had participants complete an emotional face judgment task after imagining experiencing fear-associated or disgust-associated bodily sensations. The authors found activity in the EBA for the fearful faces, when the participant had been primed with fear-associated sensations. Additionally, the functional connectivity between the EBA and a fronto-insular-temporal network was modulated based on the bodily sensations.

### Social interaction perception

So far, all studies discussed used images of single agents but EBA also plays a role in social interaction perception (for recent reviews see Abassi et al. 2024; Poyo Solanas and de Gelder 2025). Saxe et al. (2006) identified EBA as one of five regions relevant for social cognition, proposing that it provides information about the form of others’ bodies. A growing body of evidence supports this notion. For example, Sinke et al. 2010 presented participants with videos of two people either threatening or teasing each other. When participants’ attention was not explicitly drawn to the interaction, the threatening situation elicited higher EBA activity than the teasing one. Similarly, Van den Stock et al. 2015 had participants observe a violent conflict between dyads, while focusing their attention on either the aggressor or the victim. Greater EBA activity was elicited when focus was on the aggressor than on the victim. Focusing on the aggressor also elicited greater connectivity between the EBA and the STS. Quadflieg et al. 2015 had participants observe congruent (ie two agents acting in a related manner and facing each other) and incongruent interacting dyads and found that the congruency could be decoded using MVPA from the activity of the left EBA. Landsiedel et al. 2022 presented participants with images and videos of social interactions and non-social actions involving two agents. The EBA was sensitive to both motion and the interactive content. It showed greater activity for dynamic stimuli compared to static stimuli and for interactions compared to non-interactions. The results suggest the cooperation of the EBA and pSTS during dynamic interaction perception. The authors proposed that this is evidence for a third visual stream supporting dynamic social scene perception.

Experiments have investigated specifically what it is about interactions that the EBA may be processing. Walbrin and Koldewyn 2019 used MVPA to show that different types of dyadic social interactions can be decoded with above-chance accuracy from EBA activity. Additionally, EBA activity was more than simply the sum of activity for the two interaction actors presented in isolation. The EBA responses to the dyads contained unique information not included in the responses to the actors alone. Okamoto et al. 2020 conducted a study with children and adults, where they executed and observed various actions, which were varied on their initiating/following role and on the congruency of the two. Connectivity between the IFG and EBA in the initiating compared to the following condition was stronger in adults than in children. Bellot et al. 2021 used fMRI to show that the EBA responded more to stimuli of two bodies facing each other and moving toward each other than two bodies facing away from each other. Additionally, coupling between EBA and pSTS was greater for facing dyads as was also reported by Gandolfo et al. 2024. McMahon et al. 2023 utilized a large dataset of videos of dyadic interactions, which were annotated for a wide range of low- to high-level features. The authors found the EBA to be particularly involved in the representation of mid-level visual social features, such as the distance between the agents and their “facingness.”

Moreover, the EBA is involved in processing of familiarity and social knowledge. Hahn and O’Toole 2017 investigated brain regions involved in the recognition of familiar people as they approached the participants. Familiarity could be decoded from the bilateral EBA already from afar during the approach. Greven Ramsey et al. 2017 utilized connectivity analysis to show that when participants are presented with images of people they have trait-based social knowledge of, compared to images of people without associated social information, this leads to a stronger coupling between the EBA and the temporal pole, part of the theory-of-mind network.

### Action processing

The EBA plays a role in various aspects of action processing. A number of studies used adaptation paradigms to study the EBA’s involvement. For example, Kable Chatterjee et al. 2006 found that EBA activity was reduced following the repeated presentation of an action, even if the identity of the actor was novel. Wiggett and Downing 2011 used fMRI to test adaptation effects to movies of whole-body actions. While the authors did find an adaptation effect to actions and not to identities within the EBA, this effect was widespread throughout the LOTC rather than EBA specific. Kubiak and Króliczak 2016 used an fMRI repetition suppression paradigm to suggest that the left EBA was involved in processing the semantic meaning of actions with greater sensitivity for intransitive, communicative gestures.

Other methodologies have also been used to investigate EBA’s involvement in action processing. Using MVPA, Ma et al. 2018 showed that different types of movement (eg jumping, running, skipping, and walking) elicited distinct EBA activation patterns. Functional connectivity analysis further revealed that EBA interacts with a wide network of brain areas involved in behavior perception and motor control. Gu et al. 2021 utilized dynamic causal modeling to investigate relationships between the EBA, hMT+, and the pSTS during action perception, finding enhanced connectivity from hMT+ to both EBA and pSTS, as well as from EBA to hMT+ and pSTS. In addition, while much of the evidence is bilateral, some findings suggest lateralization: Romaiguere et al. 2014 found action-related activity only in the left EBA.

Several studies found the EBA to be involved in predicting action outcomes. For example, Schubotzvon Cramon et al. 2004 had participants assess the expected outcomes of actions they observed, of motor imagery they performed, or of geometrical figure sequences they were presented with. Assessing both the observed actions and the motor imagery elicited significant EBA activity, unlike the geometrical figure sequences. Abreu et al. 2012 had basketball players and novices observe basketball moves (eg free throws) and make predictions about the results. The expert players showed significantly higher EBA activity when making their predictions compared to the novices. van Elk 2014 manipulated the predictability of an action by either including or excluding actor information and by varying the number of possible target objects. The results showed that action prediction based on actor information led to greater activity in the EBA. Further support comes from the study by Takahashi et al. 2008, who found greater EBA activity for goal-directed actions compared to non-goal-directed ones. Cheng et al. 2007 reported EBA activity when participants viewed videos of food and objects being grasped, but not when the same items were presented in isolation. Similarly, Herrington et al. 2012 found that goal-directed movements resulted in stronger EBA activity than random movements, particularly when participants actively attended to the actions.

Most of the research about the EBA’s role in action processing has focused on relations between action and body perception. Hamilton et al. 2006 investigated the perceptual bias when observing another person’s action caused by the observer performing an action themselves. Participants had to judge the weight of a box from watching it being lifted, while themselves lifting boxes of varying weights. Left EBA activity was correlated with the perceptual bias and the EBA was activated by both observation of hand actions and the weight judgment task. Arzy et al. 2006 conducted an EEG study where participants were asked to imagine themselves in the position of a presented figure or they were to imagine that the figure represented their mirror reflection. Additionally, they also completed the tasks in either sitting or lying down positions. The authors found that EBA activity was related to mental embodiment of the figure as opposed to perceiving it as a mirror image. Additionally, EBA activity was also modified by the position of the participant, sitting or supine. Kuhn et al. 2011 had participants prepare to execute either manual or facial actions. Preparation for manual actions resulted in significant EBA activity and the authors proposed that this showed evidence for an ideomotor theory of action processing, where visual imagery may play a role in action control. Perhaps most convincingly, Simos et al. 2017 found the EBA to be activated by execution, observation, and imagery of finger movements.

A potential explanation for the involvement of the EBA in action perception is that it provides crucial information about body posture and kinematics to brain areas involved in action perception. In a commentary paper, Amoruso et al. 2011 proposed that information from EBA about body shape and posture is sent to a “social context network,” which is then involved in context integration with the ultimate goal of action processing. Various experimental studies have investigated this. For instance, Zimmermann et al. 2012 investigated the role of current body posture during action planning. Participants performed grasping movements while their arm posture was manipulated during trials to be congruent with the start or target posture. EBA activity was modulated by this congruency and was sensitive specifically to congruency with the goal posture, suggesting the EBA’s involvement in representing goal postures in an action plan. Zimmermann et al. 2016 conducted a TMS experiment and argued for the importance of the EBA in motor and action planning, and the EBA providing information about the state of the body. The authors found that the EBA provides structure for grasping motor plans based on current and desired states of the object and the hand and that this information is particularly important when the desired and current object configuration differ greatly. Baldassano et al. (2016) conducted two fMRI experiments to investigate brain representations of human–object interactions. Participants were presented with humans interacting with objects, humans, and objects in the same scene but not interacting, and individual humans or objects. Across all conditions, the EBA encoded information about the human pose, which was then fed to higher-order areas involved in the human–object interactions. Sasaki et al. 2018 had participants undergoing fMRI perform simple finger movements and presented visual feedback, which was varied on three dimensions: the identity of the movements (matching that performed by the hand or not), body identity (self or other), and timing (simultaneous or delayed). The left EBA was sensitive to the movement kinematics (whether the feedback matched the action), independent of body identity and feedback timing. Okamoto et al. 2021 had participants perform hand gestures, vocalizations, and facial expressions during fMRI. The participants’ actions were either preceded or followed by a congruent or incongruent action. The researchers found a strong congruency effect within the EBA for all types of actions with a stronger response for responding to actions, rather than initiating them. As such, these findings support the idea that the EBA passes on crucial information on body posture and kinematics to higher-order brain regions, which subsequently integrate this input with contextual information to enable the formation of appropriate action plans to achieve action goals.

This notion is further supported by Di Nota et al. 2016, who conducted fMRI with professional ballet dancers several times over a period of many weeks, while they learned a novel choreography. During scanning, the participants viewed and visualized the new choreography, visualized a different dance, and performed foot movements. EBA was activated most by viewing dancing compared to visualization and foot movements. Additionally, a comparison group of novice dancers showed significant EBA activity during the foot movements, while the professional dancers did not. Adamovich et al. (2009) had participants perform finger movements while they either received visual feedback about the movements through a virtual avatar or did not receive any feedback. The authors found greater EBA activity when the visual feedback was present than when it was not. van Steenbergen et al. 2017 found that preparation of a hand movement resulted in greater precision in EBA activity across participants than during preparation of a face movement. The authors suggested the involvement of the EBA to be due to the encoding of the perceptual consequences of actions during action preparation.

But there are also various studies which have argued against the EBA’s involvement in action processing. Urgesi et al. 2006 administered rTMS over the EBA and the vPMC and found that EBA stimulation impaired body form discrimination, while vPMC stimulation impaired body action discrimination, suggesting a double dissociation and the role of the EBA in processing body identity rather than action. Downing et al. 2006 presented participants with sequences of images representing an action in either a coherent order or in a scrambled, incoherent order and found greater EBA activity for the incoherent condition. The authors suggested that the EBA processes static body posture/form, rather than dynamic aspects of action. Candidi et al. 2008 applied rTMS to the vPMC and the EBA while participants decided if actions were biomechanically possible or impossible. There was no effect of EBA stimulation on any form of action processing. Piefke et al. 2009 found the EBA to be involved in the observation of face and limb movements, but not in the mental imagery of such movements. EBA responded to observation of both face and limb movements, but significantly more to the limb movements. Calmels et al. 2014 presented gymnasts with gymnastics routines, while the gymnasts were suffering from an injury and temporarily could not perform the routines. They then did this again once the gymnasts had recovered and were able to perform the movements themselves. EBA activity in response to watching the routines was independent of the participants’ own current motor repertoire. These studies thus go against the notion that the EBA is involved in action processing.

Lastly, EBA is also involved action processing in the auditory/language domain. Marsh et al. 2010 had participants read descriptions of a male college student portraying the student as either highly unlikeable, neutral, or highly likeable. The participants were then presented with action descriptions (eg “taking a drink”) and instructed to imagine the student performing the action. Together with the action description they were also presented with two other descriptors, a low-level one (eg “swallowing liquid”) and a high-level one (eg “quenching his thirst”). EBA activated highest when likeable targets’ actions were identified with high-level descriptors. de Vega et al. (2014) contrasted brain responses to factual, counterfactual, and negation sentences referencing either actions or visual events. The action sentences compared to the visual sentences resulted in greater EBA activity, independent of the type of linguistic structure.

### Development

Several studies have been conducted investigating the development of the EBA over the lifespan, particularly comparing children and adults. Pelphrey et al. 2009 compared adults with 7- to 11-year-old children and found no differences in bilateral EBA location and response profile. While Pelphrey et al. 2009 showed static images, Ross et al. 2014 presented body movement videos and found that EBA activity was stronger and the EBA size was larger in adults compared to 6- to 11-year-old children. Walbrin et al. 2020 again presented static images of bodies, but also images of social interactions to children aged 6 to 12 years and adults. Replicating the results of Pelphrey et al. 2009, there were no differences in EBA responses to bodies between children and adults, but the authors did find a part of the left EBA selective to social interactions only in the adult participants. In another study, Peelen et al. 2009 compared EBA development in a wide range of participants aged 7 to 32 years. The right EBA showed a trend of decreasing in size with age, while the left EBA showed no differences across participants. Recently, Ross et al. 2019 compared fMRI responses to happy, angry, and neutral body movements in children, adolescents, and adults. The authors found significantly higher activity in bilateral EBA for adults than for children and higher activity in right EBA for adults than for adolescents. However, the emotion modulation of the EBA remained consistent across all three age groups. Kosakowski et al. 2022 recorded fMRI data from infants between the ages of 2 and 9 months and presented them with images of faces, bodies, objects, and scenes. The authors were able to successfully localize the EBA in the infants.

EBA development has been investigated in visually impaired patients in the absence of visual input. Striem-Amit and Amedi 2014 found EBA body-selectivity even in the absence of visual experience. Using fMRI, the authors examined congenitally blind participants who perceived body shapes through a sensory-substitution process of converting images into soundscapes. The participants showed strong EBA activation for images of bodies with no such activation in temporal or parietal cortices. In another study, Kitada et al. 2014 had blind and sighted participants identify hand shapes, toy cars, and teapots using haptic information. The EBA showed greater sensitivity to hand shapes compared to inanimate objects for both blind and sighted participants. In a different line of research, Yizhar et al. 2023 investigated whether the EBA was also involved in action processing in blind participants. The authors collected fMRI data with congenitally blind participants and control participants, while they performed a variety of body movements. In the congenitally blind participants, the EBA did not show an action-related response. Additionally, their resting-state analysis showed a decreased connectivity between the EBA and sensorimotor cortices in the congenitally blind patients, with no change in the connectivity with occipital cortices.

#### Clinical disorders

The role of the EBA has also been investigated in the context of a variety of clinical disorders. Most of these studies have focused on eating disorders, but there have also been studies on other diagnoses, such as major depressive disorder, Parkinson’s disease, schizophrenia, autism, Alzheimer’s disease, and anxiety disorders.

#### Major depressive disorder

Paul et al. 2019 investigated the relationship between the EBA and the default mode network patients with major depressive disorder (MDD), finding that reduced connectivity between these regions predicted higher levels of depersonalization symptoms. This suggests that disruptions in body-related processing may contribute to the experience of disconnection from oneself, a hallmark of MDD. Haipt et al. 2022 expanded on this by using fNIRS to measure brain activity during an emotional human gait paradigm in both depressed patients receiving either hypnotherapy or cognitive-behavioral therapy, and healthy controls. Patients undergoing hypnotherapy showed a decreased functional connectivity between the EBA and the STS. This was specifically due to a decrease in STS activation for hypnotherapy patients, who also reported less ruminating. This suggests that hypnotherapy interventions may modulate the neural networks involved in body perception and emotional processing in MDD, which includes the EBA.

#### Parkinson’s disease

Research into Parkinson’s disease (PD) has highlighted the EBA’s potential role in compensatory motor processes, particularly when motor functions are disrupted. Helmich et al. 2007 explored this idea by having PD patients with right-lateralized symptoms complete a mental hand rotation task. The researchers found greater right EBA activity for judgments involving the more affected right hand. This was matched by greater connectivity between the right EBA and the left dPMC. The authors thus suggested that the EBA was recruited as part of a compensatory mechanism in the strongly lateralized patients. Building on this, van Nuenen et al. 2012a used brain stimulation methods to further explore the role of the EBA in PD patients. In their study, the authors applied cTBS to inhibit the EBA and the dPMC in PD patients as well as control subjects while they completed a mental hand rotation with either congruent or incongruent hand positions. The posture congruency effect was lost in PD patients following stimulation of the EBA, but not in the control patients, while in the control patients the congruency effect was reduced during stimulation of the dPMC, but not in the PD patients. The authors suggested that in PD, the EBA compensates for the dysfunction in the dPMC to support functions.

In another study, van Nuenen et al. 2012b examined presymptomatic individuals with a genetic predisposition for developing PD. Participants and controls completed a motor imagery task while undergoing fMRI. In the genetic mutation carriers, greater striatal impairment was associated with stronger effective connectivity between the right premotor cortex and the right EBA. The authors thus proposed that the absence of PD symptoms in those participants may be due to compensatory mechanisms involving the EBA.

#### Autism spectrum disorder

A series of studies has also investigated the potential involvement of the EBA in autism spectrum disorder (ASD), looking at both EBA function and anatomy. For example, Okamoto et al. 2014 asked participants with ASD and neurotypical controls to both execute and observe movements, manipulating the congruency and the order of these actions. The authors found a congruency effect in the bilateral EBA for both groups. However, when action preceded observation, there was a reduced congruency effect in the left EBA of the ASD group compared to the control group. In a subsequent study, Okamoto et al. 2017 compared children and adults with ASD and control participants on the perception of faces, bodies, cars, and scenes using fMRI. While adult EBA responses and size were no different between ASD participants and controls, the EBA was smaller in ASD children compared to typically developing children. Further investigating body perception, Okamoto et al. 2018 examined how participants with ASD and typically developing individuals perceived their own and others’ hands from an allocentric and egocentric perspective. Patients with ASD showed a significantly smaller difference between egocentric and allocentric perspectives in left EBA activity than the control subjects, indicating a possible disruption in self–other distinction at the level of perceptual encoding. These findings thus all suggest an involvement of the EBA in ASD.

#### Schizophrenia

Takahashi et al. 2010 investigated EBA function in schizophrenia by presenting patients and age- and sex-matched controls with sports-related motions in congruent and incongruent contexts. Compared to the controls, the schizophrenia patients had significantly lower activity in the EBA for context-congruent actions. Moreover, the EBA activity in patients was negatively correlated with the severity of psychopathology symptoms. The authors suggested that EBA dysfunction may lead to impairments of simulation, learning, and execution of actions and movements. In a follow-up study, Takahashi et al. 2012 showed that EBA activity could be modulated through intervention. Schizophrenia patients completed a 3-month exercise program a control patient group did not participate in. The patients who had undergone the sports program showed greater EBA activity when observing sports-related actions and this greater activity was related to improvement on a Positive and Negative Syndrome Scale. These results suggest a potential link between exercise, EBA engagement, and symptom reduction. For a previous systematic review highlighting the involvement of the EBA in schizophrenia, see van der Stouwe et al. 2018.

#### Eating disorders

The involvement of the EBA in eating disorders has been extensively studied, with investigations into anorexia nervosa, binge eating disorder, and bulimia. Suchan et al. 2010 found that patients with anorexia nervosa (AN) have a significant reduction in gray matter density in the left EBA compared to controls. Building on this, Suchan et al. 2013 investigated functional connectivity during body perception tasks. When viewing images of bodies versus chairs, AN patients and control participants showed significantly different patterns of connectivity between early occipital regions, the EBA, and the FBA. In fact, the effective connectivity between the left EBA and FBA in the AN patients showed a negative correlation with body-size misjudgment scores. In a different study, Vocks et al. 2011 found that EBA activity in AN patients was modulated by satiety: patients showed greater EBA activation when drinking chocolate milk while satiated compared to when hungry, indicating a possible interaction between interoceptive states and body-related processing in the EBA.

The use of body images of the self and others with various weight and size distortions has been a popular paradigm in experiments involving AN patients, although yielding varied results. In a review of neuroimaging studies about self-perception in AN, Esposito et al. 2018 concluded that the EBA was one of the areas showing activity modulations during body image perception and body-size estimation tasks. Supporting this, Vocks et al. 2010b found increased EBA activity in AN patients following a body-image therapy involving images of their own bodies. Kodama et al. 2018 presented participants who had recovered from AN and control subjects with normal and weight-distorted images of their own and others’ bodies. Compared to the control subjects, the recovered AN patients showed significantly lower EBA activity in response to images of their own bodies. In contrast, Castellini et al. 2013, using a similar paradigm with patients currently suffering from AN, found no differences between the AN patients and controls in EBA activation. Overall, the research suggests that anorexia nervosa affects the functional, connectivity, and density properties of the EBA, although the exact function the region plays remains to be elucidated.

Furthermore, Vocks et al. 2010c had patients with anorexia and bulimia nervosa undergo fMRI before and after cognitive-behavioral body-image therapy, as well as without having undergone any treatment, while viewing images of their own and others’ bodies. There was stronger activity in the EBA when participants observed their own bodies after treatment compared to before treatment. Mattavelli et al. 2019 applied tDCS to the right EBA, mPFC, or sham stimulated while patients with anorexia and bulimia nervosa and control subjects completed implicit association tests on the images of food, bodies, flowers, and insects. Stimulation of the EBA resulted in weaker preference for tasty food only in the eating disorder patients and not in the control participants. Moreover, Lavagnino et al. 2014 investigated functional connectivity alterations in bulimia nervosa patients, specifically. BN patients had significantly decreased functional connectivity between the somatosensory network and the EBA compared to control participants.

Press et al. 2022 investigated neural responses in patients with binge eating disorder (BED), as well as healthy controls, using fMRI and simultaneous eye-tracking to assess visual attention. Participants viewed images of their own bodies, weight-matched bodies, and non-body stimuli. Visual attention to the presented stimuli as measured with eye-tracking was related to higher EBA activity across both groups. However, it was the FBA that showed significantly greater activity in the BED group compared to the control group during body-image viewing. In a follow-up study, Press et al. 2023 presented participants with BED and control participants with body-part images of their own bodies and others’ bodies and asked them to rate the attractiveness of each. The authors found significantly higher activity in the insula and amygdala for negatively valenced images of their own bodies, but there were no differences in EBA activity.

#### Other clinical conditions

Some additional studies into the role of the EBA in various clinical states have been conducted. This includes studies into EBA activity in Alzheimer’s disease (AD), anxiety disorders, and prosopagnosia. For instance, Canario et al. 2023 investigated EBA activity in patients with AD and a group of control subjects. There were no clear differences in EBA responses between the two participant groups. Kikuchi et al. 2017 used positron emission tomography to measure brain cerebral glucose metabolic rates while participants with varying rates of cognitive decline performed a mental hand rotation task. The glucose metabolism level in the right EBA was significantly correlated with task performance. Additionally, applying tDCS to the right EBA to younger, healthy participants resulted in a significant improvement in task performance.

Grossi et al. 2017 conducted functional connectivity analysis on resting-state fMRI data on participants with illness anxiety disorders (IADs) and healthy controls. Compared to controls, patients with IADs exhibited reduced connectivity between the left EBA and the paracentral lobule. Additionally, behavioral questionnaire results correlated with hyper-connectivity between the EBA and amygdala and hippocampus. Brown et al. 2019 investigated brain regions involved in self-referential processing in participants with social anxiety disorder (SAD) before and after therapy and healthy controls. Greater SAD severity was related to greater activation for self-versus-other processing in the left EBA.

Van den Stock et al. 2008 found that patients with developmental prosopagnosia showed higher activation for neutral faces in the EBA compared to control participants, suggesting that face-sensitive processes are less segregated within the brain in prosopagnosic patients.

#### Neuroaesthetics

The EBA has also been implicated in studies investigating the neuronal bases of aesthetic judgments, particularly judgments about bodies. Many of the studies utilized NIBS techniques to directly investigate the role of the EBA (see Cattaneo 2020 for a review). There has also been an fMRI study, where Holliday et al. (2011) had participants rate the attractiveness of computer-generated female bodies systematically varied in BMI, but not body shape, with BMI changes modulating activity within the EBA.

Calvo-Merino et al. 2010 showed that rTMS over the EBA led to a change in participants’ aesthetic sensitivity to body stimuli, while rTMS over the vPMC did not show any effect, nor was there an effect of any stimulation on control stimuli judgments. Applying rTMS over the left EBA, right EBA, and the vertex, Cazzato et al. 2014 found that for women, rTMS over the right EBA led to more positive aesthetic judgments for bodies of the opposite gender. On the other hand, for men, rTMS over both left and right EBA resulted in fewer positive aesthetic judgments of the opposite gender. Cazzato et al. 2016 applied rTMS over the EBA, which led to increased positive aesthetic judgments for bodies of the opposite gender to the participants. Finally, Cazzato et al. 2017 applied cathodal tDCS stimulation over the left EBA, which diminished the extent of a weight bias in male participants compared to sham stimulation. These studies thus suggest that the EBA is involved in making aesthetic judgments.

#### Multisensory processing

There has also been a series of studies investigating the EBA’s involvement in multisensory processing, both in the context of multisensory integration and separately as a modality-independent categorical area. Beer et al. 2013 had participants observe lip or body movements, hear speech or body action sounds, or a combination of both. The EBA was engaged during bimodal stimulation, with a subadditive response for the bimodal compared to the unimodal stimuli. Kim et al. 2019 used MVPA to show that perceived hardness from haptic and visual information was being encoded by bilateral EBA and suggested that this may be due to the finger movements involved in the visual perception of hardness.

Conversely, Fourcade et al. 2022 found that the EBA did not differentiate between congruent and incongruent haptic stimulation of the arm, but it did show higher activity for an aversive (spider) condition compared to a neutral (toy car) condition. Similarly, Atilgan et al. 2023 administered cTBS over the left EBA, which led to a disruption of performance for visually identifying hands and teapots (with no effect on cars) compared to other stimulation sites and haptic identification.

#### Functional near-infrared spectroscopy

While most studies investigating the EBA used either fMRI or NIBS, a smaller number of studies used other methodologies, including functional near-infrared spectroscopy (fNIRS), electroencephalography (EEG), and magnetoencephalography (MEG).

Ishizu et al. 2009 conducted a series of fNIRS experiments, in which they recorded EBA activity when participants were shown images of human bodies, performed simple hand movements, and imagined performing hand movements. The researchers found increased EBA activity for perception of bodies, execution of movements, and imagination of movements. Shimada 2013 was able to localize the EBA in 11 out of 16 subjects using fNIRS. Schneider et al. 2014 found greater EBA activity for negative emotional gaits compared to neutral ones, and the authors also found the activity to be greater when attention was paid directly to the emotions rather than to the walking speed.

### EBA time course and oscillations

#### Electroencephalography

EEG studies using body images and body parts have identified a variety of event-related potentials (ERPs) thought to originate in EBA activity. Body images are processed as rapidly as face images as shown by body-specific activity in the N170 window (Stekelenburg and de Gelder 2004). Mado Proverbio et al. 2008 found a significantly larger amplitude of an N2 component (210 to 270 ms post-stimulus) for images of people compared to scenes, which originated in the right EBA. The authors only found this effect in their female participants. Möhring et al. 2014 also found an N250 component originating in the right EBA, using a paradigm of repeated presentation of rock-paper-scissors gestures. This N250 component showed adaptation for repetition of all gestures, and an N190 component showed repetition adaptation for repetitions of the same gesture.

Taylor et al. 2010 found that the event-related potential marker N1 showed similar patterns of stimulus selectivity as the EBA, suggesting the EBA as the source of the component. Sadeh et al. 2011 applied TMS stimulation over the EBA, resulting in an increased amplitude of the N1 (150 to 200 ms post-stimulus onset) component elicited by images of bodies only and not for images of faces. Espirito Santo et al. (2017a) found a stronger N1 component (170 ms after stimulus) originating from occipitotemporal electrodes for distorted hands compared to natural hand configurations. Espirito Santo et al. (2017b) found this N1 component to show differences in amplitude for images of hands versus bodies. Moreau et al. 2017 presented participants with images of fingers, hands, arms, and whole bodies, as well as control plant images. Occipitotemporal electrodes showed a larger N190 component for the body images compared to the control images. Additionally, for the same electrodes, there was a theta-band event-related synchronization specific for images of hands and arms. Moreau et al. 2020b once again showed an occipitotemporal N1 component with higher amplitude for images of hands compared to the non-body images. The hand images also resulted in a greater theta-band power around the occipitotemporal electrodes with higher theta for identified compared to unidentified hands as well. Costa et al. 2023 examined ERPs from professional tennis players while they observed images of tennis players and had to predict the ball’s landing position. Additionally, in some images different parts of the body were occluded. Occlusion of the trunk led to the worst performance accuracy and response time and additionally led to a lower amplitude of the N1 component in the right hemisphere in the occipitotemporal regions.

Other components have also been identified by studies with widely varying tasks. Ortigue and Bianchi-Demicheli 2008 had participants complete a sexual desire decision task with images of high and low desirability. This elicited several relevant visual event-related potentials (P100, N200, and P300) and both desirable and non-desirable stimuli showed evidence of EBA recruitment. Mado Proverbio et al. 2012 presented naïve participants and professional basketball players with correct and incorrect basketball movements. Only the skilled participants (professional basketball players) showed a significant N400 response for incorrect actions with the response involving the EBA. Giabbiconi et al. 2016 recorded and analyzed steady-state visually evoked potentials (SSVEPs) by recording EEG signal while presenting participants with images of bodies, chairs, and non-objects. Source reconstruction showed an SSVEP in the lateral occipitotemporal areas elicited only during the presentation of bodies. Quinzi et al. 2019 recorded greater bilateral occipitotemporal activity, likely originating from the EBA, for externally triggered movements compared to self-originating ones. The authors suggested that this may be anticipatory activity reflecting a visual prediction of the goal posture. Caggiano et al. 2022 presented participants with images of hands and feet and investigated if recognition of the body parts was facilitated by first briefly presenting functionally related objects. The results show that related primes led to much more accurate responses than unrelated ones. Additionally, the primes modulated a posterior N200 and a centro-parietal P300 component.

Finally, two studies have looked specifically at oscillatory activity related to the EBA. Moreau et al. 2020a had participants synchronize their movements with those of a virtual avatar they were observing. On some trials, the avatar would unexpectedly change their movement, creating a prediction error for the participant. The authors found this prediction error to be associated with theta- and alpha-band modulations in frontocentral electrodes, and additionally, this theta and alpha activity was in phase with theta and alpha at occipitotemporal electrodes. Salgues et al. 2021 investigated the role of the EBA in spontaneous sensations, a phenomenon related to self-awareness and interoception. There was greater EBA and TPJ functional connectivity in the alpha and theta bands related to less frequent spontaneous sensations.

#### Magnetoencephalography

MEG studies have focused primarily on investigating EBA responses to visually presented body-related stimuli with one study also investigating the EBA’s involvement in the sense of agency.

Ishizu et al. 2010 showed activity in response to presentation of body images in the bilateral EBA, originating about 190 ms post-stimulus onset and with a right-hemisphere dominance. Ishizu 2013 then presented participants with meaningless blobs, while suggesting interpretation of the blobs as images of bodies in the participants. Perception of the blobs as bodies was correlated with EBA activity, which was preceded by significant IFG activation, suggesting a top-down effect. Meeren et al. 2013 recorded body-selective MEG responses at 120 to 200 ms, constrained to the lateral occipitotemporal cortex, which includes the EBA, and afterwards spreading to the orbitofrontal cortex and then ventral temporal cortex. The authors highlighted the role of the EBA in early body perceptual processes. Nakamura et al. 2015 used MEG and presented participants with images of feet, hands, mouths, and objects. The body-part categories could be decoded from the resulting EBA activation with significant above-chance accuracy.

Buchholz et al. 2019 investigated the brain representations of a sense of agency by manipulating causal belief during a visuo-motor tapping paradigm. When participants experienced a sense of agency, this led to a disappearance of local feed-forward activity within the EBA, as measured by gamma-band power.

#### Subdivisions of EBA

There have been suggestions from some research that the EBA is not one unified area but is instead composed of several subdivisions. For instance, when comparing responses higher for body parts than objects, Astafiev et al. 2004 identified two peaks in the lateral occipital cortex, a dorso-anterior EBA and a ventro-posterior EBA. Spiridon et al. (2006) identified two parts to the EBA, one anterior to the MT/V5 region and one posterior to it. Kubiak Kroliczak et al. 2016 found that repetition suppression for intransitive gestures as opposed to transitive gestures occurred only in a rostral part of the EBA.

Next to this, Weiner and Grill-Spector 2011 investigated responses to images of limbs as well as localizing the human motion-selective complex (hMT+) and found three limb-selective regions surrounding the hMT+ in a crescent rather than a single limb-selective area. One area was posterior to the hMT+ on the lateral occipital sulcus/middle occipital gyrus (LOS/MOG), another was anterior to hMT+ on the middle temporal gyrus, and a third was inferior to hMT+ on the inferotemporal gyrus. This was consistent over a period of 3 years and independent of images presented. There were different preferences regarding image positions amongst the three regions.

Furthermore, Ferri et al. 2013 used functional localizers to investigate EBA overlap with the retinotopic MT/V5 area and its four subregions. Each of the four retinotopic MT/V5 areas included EBA voxels, although the majority of EBA voxels were outside the MT/V5 clusters. Additionally, the main locus of EBA voxels was rostrally outside the MT/V5 region. The authors ascribe the differences in findings compared to Weiner and Grill-Spector 2011 primarily to differences in hMT+ and EBA localizers. Finally, Li et al. (2023) related subdivisions of EBA to behavioral relevance found four adjacent body-selective areas within the LOC network showing distinct connectivity profiles and functional roles.

## Part II—Coordinate review

Numerous studies identified in the literature review have reported activation in the EBA and have associated the region with a variety of cognitive functions, but there has been no systematic comparison of the anatomical labels and coordinates associated with the EBA across the literature. Individual studies often identify the EBA, but the reported locations can vary considerably due to differences in stimuli, experimental design, analysis pipelines, and participant sample characteristics. To qualitatively assess the spatial consistency of EBA location, we extracted the reported anatomical locations in these papers. Next, we quantitatively assessed the consistency of EBA localization by compiling activation coordinates from these studies and conducted an activation likelihood estimation (ALE) meta-analysis using data retrieved from NeuroSynth, a framework designed for conducting large-scale neuroimaging meta-analyses (Yarkoni et al. 2011). This approach allowed us to statistically integrate results from multiple experiments and generate a probabilistic map of the brain regions most consistently reported as the EBA, providing a comprehensive and reproducible reference for its anatomical location.

### Methods

#### EBA coordinate extraction from literature review articles

From all the articles included in the literature review (Part I), we extracted EBA coordinates when available. This included both EBA coordinates from individual participants as well as group coordinates resulting from statistical analyses. When coordinates were reported in Talairach space, they were converted to MNI coordinates using the BioImage Suite online tool (https://bioimagesuiteweb.github.io/webapp/mni2tal.html), which applies the mapping developed by Lacadie et al. 2008. This resulted in 768 sets of coordinates extracted from 133 research articles.

#### ALE meta-analysis

**Figure 3:**
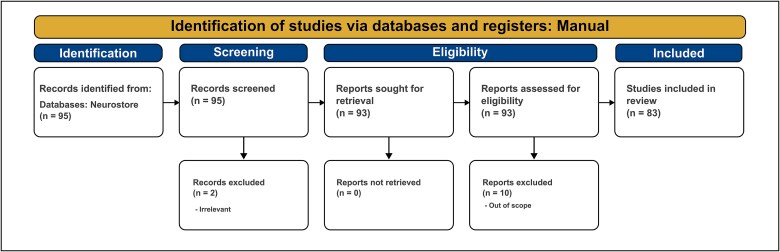
NeuroSynth review PRISMA diagram. Diagram explaining the selection of articles included in the NeuroSynth coordinate review. After the initial record identification, two articles were excluded based on review of their abstracts for not being related to the EBA. Subsequently, 10 more articles were excluded as the EBA was mentioned only tangentially and was not included in the results of the papers.

An ALE meta-analysis (Eickhoff et al. 2012; Turkeltaub et al. 2012, 2022) was performed with NiMARE 0.5.2 (Salo et al. 2023) using an ALE kernel. An ALE kernel (Eickhoff et al. 2012) was used to generate study-wise modeled activation maps from coordinates. In this kernel method, each coordinate is convolved with a Gaussian kernel with a full width at half maximum of 8 mm. For voxels with overlapping kernels, the maximum value was retained. ALE values were converted to *P* values using an approximate null distribution (Eickhoff et al. 2012). The input dataset included 2,723 foci from 83 experiments (see Fig. 3). False discovery rate was performed with the Benjamini–Hochberg procedure (Benjamini and Hochberg 1995).

### Results

#### Experimental paradigms for EBA localization

Of the 233 articles included in the literature review, 189 localized the EBA with their own stimuli while others took coordinates from previous studies or identified the region post hoc. The most common stimuli and contrast used were bodies and chairs (9.5% of studies). Also, 52.4% of the studies utilized static images of bodies in contrast with a variety of other stimuli (including faces, scenes, flowers, tools, textures, mammals, and other objects). Another 19.0% included static images of body parts also contrasted with a wide range of other stimuli. The remaining 28.6% of the studies used different stimuli to identify regions termed EBA by the authors. These included basketball-related motions, tests assessing knowledge about body parts, exteroceptive arm positions, videos of dyadic interactions, gymnastics routines, emotional gait patterns, mental imagery of body movements, images of participants’ own bodies, point-light displays, grasping movements, speech and body sounds, words associated with actions toward and away from the body, and many others.

#### Anatomical labels across the literature

Some authors localized the EBA to one or more anatomical brain structures. This included the middle occipital gyrus (Brown et al. 2019; Calmels et al. 2014; Corradi-Dell Acqua et al. 2015; Felician et al. 2009; Helmich et al. 2007; Kitada et al. 2014; Limanowski and Blankenburg 2016; Limanowski et al. 2014; Morris et al. 2006; Rueschemeyer et al. 2010, van de Riet et al. 2009; Van den Stock et al. 2015), middle temporal gyrus (Corradi-Dell’Acqua et al. 2015; de Vega et al. 2014; Helmich et al. 2007; Kitada et al. 2014; Marsh et al. 2010; Romaiguère et al. 2014; van de Riet et al. 2009; Willems et al. 2010), middle occipital lobe (de Vega et al. 2014), occipitotemporal cortex (Kim et al. 2019), supramarginal gyrus (Kitada et al. 2014), superior temporal gyrus (Kitada et al. 2014), inferior temporal gyrus (Kitada et al. 2014), fusiform gyrus (Kitada et al. 2014), angular gyrus (Kitada et al. 2014), inferior occipital gyrus (Kitada et al. 2014), superior occipital gyrus (Kitada et al. 2014), superior temporal cortex (Moro et al. 2008), inferior occipitotemporal cortex (Moro et al. 2008), middle occipitotemporal cortex (Moro et al. 2008), lateral occipitotemporal cortex (Romaiguere et al. 2014), lateral temporal cortex (Saygin et al. 2011), and lateral occipital cortex (Van de Vliet et al. 2018).

#### Coordinate distribution across the literature

**Figure 4:**
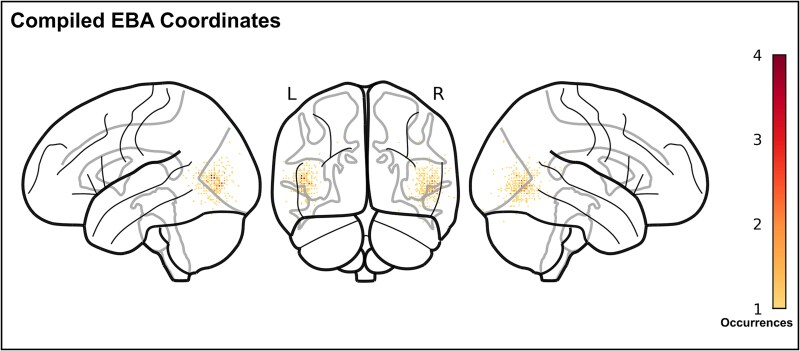
EBA coordinates compiled from literature review. A total of 768 sets of EBA coordinates were extracted from 133 articles included in the literature review. Coordinates are both from individual subjects and as group results from statistical analyses. The colorbar indicates the number of repetitions of a given coordinate set.

Similarly to the diversity of anatomical locations associated with the EBA, the reported coordinates also span a wide region of the brain across the occipital and parietal lobes (see Fig. 4). Fifty-five coordinate sets were reported more than once, with six coordinates occurring three times in the dataset and two coordinates occurring four times. In the left hemisphere, 339 sets of coordinates were reported, averaging (−48.1, −71.6, 4.4) and ranging widely across spatial dimensions (*x*: −64 to −22, *y*: −92 to −48, *z*: −20 to 36). In the right hemisphere, 429 sets of coordinates were reported, averaging (49.1, −68.1, 2.4) and again showing substantial variation (*x*: 21 to 72, *y*: −98 to −34, *z*: −26 to 37).

The coordinates in each hemisphere were also averaged per research topic as identified in the literature review (see Table 1).

**Table 1:** Average EBA coordinates per research topic.

	Left hemisphere				Right hemisphere			
	Count	*x*	*y*	*z*	Count	*x*	*y*	*z*
Action processing	34	−48.3 (4.3)	−69.7 (3.3)	4.1 (5.8)	30	49.3 (6.9)	−66.5 (6.5)	0.6 (5.1)
Body ownership	21	−47.7 (7.4)	−64.8 (5.9)	8.1 (5.8)	21	50.4 (5.6)	−65.5 (8.1)	3.9 (4.6)
Body-selectivity	62	−47.4 (6.1)	−71.1 (3.6)	3.1 (4.4)	142	47.3 (6.1)	−68.4 (5.1)	6.0 (5.8)
Clinical	75	−47.8 (8.0)	−70.8 (6.2)	0.8 (5.1)	84	47.9 (6.7)	−67.6 (5.9)	5.8 (5.6)
Connectivity	1	−49.0 (−)	−60.0 (−)	10.0 (−)	4	54.8 (10.9)	−75.3 (10.0)	−11.8 (17.4)
Development	15	−45.9 (6.0)	−69.4 (10.0)	8.4 (6.1)	16	48.1 (11.5)	−64.8 (13.1)	8.1 (12.3)
EEG	1	−49.0 (−)	−60.0 (−)	10.0 (−)	0	…	…	…
Emotion perception	35	−52.7 (4.7)	−67.6 (8.7)	6.9 (5.5)	31	50.5 (8.9)	−66.0 (5.6)	5.1 (6.4)
fNIRS	0	…	…	…	11	56.3 (9.0)	−74.8 (8.6)	10.0 (17.4)
MEG	1	−49.0 (−)	−60.0 (−)	10.0 (−)	1	57.0 (−)	−83.0 (−)	7.0 (−)
Multisensory processing	27	−46.7 (8.5)	−67.9 (10.0)	4.9 (8.7)	32	50.6 (6.3)	−65.4 (9.1)	4.3 (7.6)
Neuroaesthetics	2	−49.0 (3.3)	−71.5 (0.7)	0.5 (0.7)	2	47.5 (10.6)	−73.0 (1.0)	−1.0 (1.0)
Self vs. other	23	−49.5 (6.1)	−65.3 (5.8)	4.5 (5.4)	43	48.3 (7.1)	−64.5 (5.4)	2.7 (4.4)
Social interaction perception	31	−45.5 (4.9)	−75.2 (6.4)	8.1 (8.1)	9	45.4 (6.1)	−75.5 (8.1)	5.4 (9.8)

“Count” columns indicate the number of coordinate sets per research topic and brain hemisphere. For the coordinates, the numbers in brackets indicate standard deviations.

#### ALE meta-analysis

**Figure 5:**
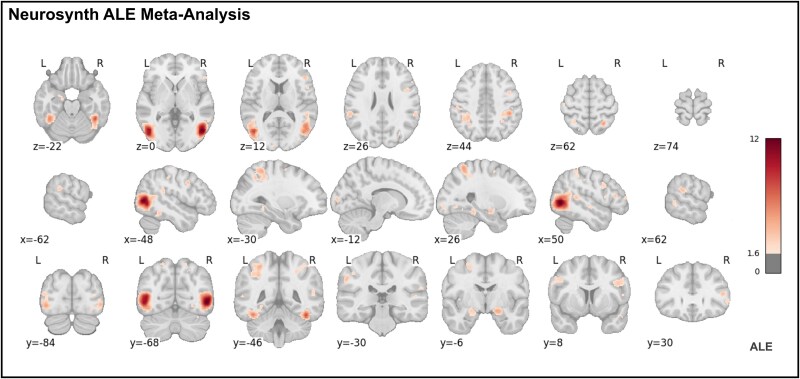
NeuroSynth ALE meta-analysis results. Brain regions showing significant ALE meta-analysis values based on a literature search for the EBA. Coordinates are in MNI space.

The results of the ALE meta-analysis identified two clusters in bilateral occipitotemporal regions (see Fig. 5). The left hemisphere cluster had a peak voxel at (−48, −72, 4) and a cluster size of 15,368 mm^3^, while the right-hemisphere cluster had a peak voxel at (50, −68, 0) and a cluster size of 23,232 mm^3^. The meta-analysis also identified another 29 clusters throughout the brain.

## Discussion

The average coordinate locations identified by the two approaches were similar for both coordinate reviews, while the wide range in individual coordinate sets was likely due to the different stimuli and tasks used to localize the EBA across the studies. Although reported anatomical labels and coordinates are heterogenous, the ALE meta-analysis provides converging evidence for bilateral occipitotemporal clusters, reinforcing the robustness of the EBA as a functional region. This systematic reference forms the basis for comparison with our own localizer experiment. This localizer utilized highly controlled data collection and analysis methods to contrast the EBA identified under these strict conditions against the previous wide-ranging results.

## Part III—Functional localizer

The results of the two coordinate reviews converged on the EBA being represented in a wide region of the brain around the lateral occipitotemporal cortices. While the average coordinate locations were similar for both coordinate reviews, the wide range was likely due to high variability in stimuli and tasks used to localize the EBA across the studies. With the coordinate reviews serving as a new ground truth for EBA location, we aimed to confirm these results with a new collection of fMRI data obtained with highly controlled acquisition and analysis parameters and protocols.

As the results of Parts I and II showed, more than 70% of the studies included used an EBA localizer consisting of static images, even though dynamic body stimuli result in greater EBA activation. Additionally, many localizers compared body stimuli with one other semantic category (eg chairs) as a control object. This warrants the development of a new localizer, which could be directly contrasted with the results of the meta-analysis findings, further exploring the differing EBA responses throughout the posterior brain. This would allow us to investigate how different stimuli affect the resulting EBA localization. Building on the identified localizers in existing research, our localizer experiment utilized dynamic stimuli and compared body stimuli with two other semantic categories (faces and objects), as well as scrambled versions of all the stimuli to control for low-level visual properties.

### Methods

#### Participants

Nineteen healthy participants (mean age = 27; age range = 23 to 32; 10 male, 9 female) completed the localizer runs. All participants had normal or corrected-to-normal vision and no history of psychiatric or neurological disorders. The task was explained to all participants, and they provided written consent before they underwent the scanning. Participants received gift vouchers as a participation reward. The experiment was approved by the Ethics Review Committee Psychology and Neuroscience (ERCPN) at Maastricht University (ERCPN-188_03_02_2018) and was conducted in accordance with the Declaration of Helsinki.

#### Stimuli

The participants were presented with stimuli depicting 1-s-long (60 frames/s) grayscale videos of bodies, faces, objects, and their scrambled counterparts. The bodies and faces images were first used in Kret et al. (2011b). The images displayed naturalistic full-body or facial movements, respectively. The actors wore black clothing and were shown against a greenscreen background. The actions consisted of angry, fearful, happy, and neutral behaviors. In the body stimuli, the actors’ faces were blurred. The objects consisted of synthetic moving objects with the video aspect ratio matched to the body videos. They were first used in a study by Li et al. (2023). The size of the stimuli was 3.5 * 3.5 degrees of visual angle for the faces and 3.5 * 7.5 degrees for the bodies and objects. The original background of the videos was removed and replaced with a dynamic white noise background spanning 17.23 * 10.38 visual angle degrees. The dynamic white noise was made up of squares of 3 by 3 pixels in which the grayness level was randomly sampled from a uniform distribution at a rate of 30 Hz. To control for low-level visual feature differences among the three categories (bodies, faces, objects), mosaic-scrambled versions of the videos were also included as stimuli. This scrambling preserved local motion, luminance, contrast, non-background area, but disrupted the overall shape of the stimuli and the global motion. This led to a total of six experimental conditions (body, face, object * normal, scrambled; see Supplementary Figs. S1 to S4 for examples). There were 10 different stimuli per condition, equaling 60 unique videos.

#### Procedure

Participants completed two runs of the functional localizer paradigm (subject 01 only completed one run). The runs were structured in a block-design, where participants saw ten 1-s videos showing one of the six types of stimuli (faces, bodies, objects * scrambled, normal) in each block and 10 different stimuli per stimulus type. The order of the blocks was randomized for each participant. Participants were asked to stay focused on a fixation cross overlaid on top of the stimuli throughout each run. Per run, each block of stimuli was presented three times, and an additional catch block was included, where the participant had to press a button with their index finger of the right hand when the fixation cross became a circle. The response was not conditional on the stimulus, only the fixation cross. Stimuli were presented for 1 s with an inter-stimulus interval of 1.5 s and a jittered inter-block interval (8.5, 10.5, or 12.5 s). The stimuli were presented, and responses recorded using MATLAB (version R 2021b) with PsychToolbox (v3; Brainard 1997, Pelli 1997, Kleiner et al. 2007). The whole experiment lasted ~15 min. The participants also underwent MRI scanning for another experiment during the same session, the results of which are not reported here.

#### fMRI acquisition

Scanning was conducted using a 3-T Magnetom Prisma Fit scanner (Siemens Healthineers, Erlangen, Germany) with a 64-element head–neck coil at the Maastricht Brain Imaging Centre, Maastricht University, the Netherlands. The functional images were acquired with a T2*-weighted 2D echo-planar image sequence (number of slices per volume = 56, no gap, 2 mm isotropic resolution, repetition time (TR) = 1,300 ms, echo time (TE) = 23 ms, flip angle (FA) = 68, field of view (FoV) = 1,600 × 16,000 mm^2^, matrix size = 800 × 800, multiband acceleration factor = 4). The number of volumes collected per run was 380 with a total scan time per run of 8.1 min. A three-dimensional MPRAGE imaging sequence (Mugler and Brookeman 1990) was used to obtain high-resolution structural images for each participant (1 mm isotropic resolution, TR = 2,300 ms, TE = 2.98 ms, FA = 9, FoV = 256 × 256 mm^2^, matrix size = 256 × 256, total scan time = 6 min).

#### fMRI analysis

The data preprocessing and analysis were conducted in BrainVoyager. The functional runs underwent slice scan time (cubic spline) and motion (sinc interpolation) correction and were also temporally filtered (high-pass filter: 0.006 Hz). One subject exhibited significant motion artifacts which could not be reliably corrected, so their data were removed from further analysis, leaving 18 subjects. The anatomical data first had brain extraction applied using the default BrainVoyager pipeline and then intensity inhomogeneity correction (sinc interpolation). The functional runs were then spatially aligned to the anatomical data, and everything was normalized into MNI space (Fonov et al. 2009; Fonov et al. 2011).

For each functional localizer run, a design matrix was created, which included predictors for each of the six stimulus categories (body, face, object; scrambled, non-scrambled), as well as *z*-transformed motion parameters derived from the preprocessing. The design matrix was then used to calculate a general linear model (GLM) for each run. Volume maps were created for two predefined comparisons (body unscrambled vs. body scrambled and faces and objects unscrambled, body unscrambled vs. body scrambled). Any resulting voxel clusters were corrected for multiple comparisons using the Cluster-Level Statistical Threshold Estimator plugin within BrainVoyager (5,000 iterations, alpha level = 0.05, initial *P* = 0.001). The design matrices were also combined to run a multisubject GLM. For this GLM, the same procedures were followed as for the single-subject GLMs, except the volume time-course data were first spatially smoothed using a Gaussian filter with a full width at half maximum of 4 mm. The same volume map contrasts were then created from the multisubject GLM, also cluster-level corrected using the same parameters.

Finally, for each subject, surface meshes were created using the BrainVoyager automatic cortex gray- and white-matter segmentation pipeline. Volume maps were then projected onto the cortical surface of the gray matter using trilinear interpolation and sampling the volume at the folded cortex vertex positions to create the surface maps.

### Results

#### Multisubject analysis

**Figure 6:**
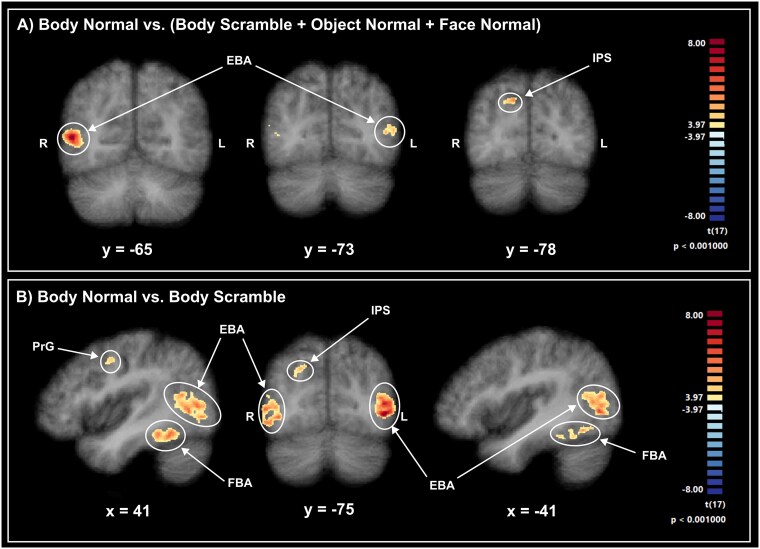
Statistical maps of multisubject contrast results. (A) Clusters identified in the multisubject analysis showing significantly higher activity for videos of normal bodies compared to scrambled bodies, normal objects, and normal faces. (B) Clusters identified in the multisubject analysis showing significantly higher activity for videos of normal bodies compared to scrambled bodies. Details about all the regions can be found in Table 1. Coordinates are in MNI space. Abbreviations: EBA, extrastriate body area; IPS, intraparietal sulcus; PrG, precentral gyrus; FBA, fusiform body area.

The strict contrast of normal bodies versus scrambled bodies, normal faces, and normal objects revealed three clusters showing greater activity for normal bodies compared to the other three conditions. These were the bilateral LOTC and the right intraparietal sulcus. Additionally, a less strict contrast of normal bodies versus scrambled bodies resulted in seven clusters showing significantly greater activation for the normal bodies. These consisted of the bilateral LOTC, the right precentral gyrus, the right entorhinal cortex, and the right intraparietal sulcus (see Table 2 and Fig. 6).

**Table 2:** Multisubject GLM results for body-selective regions.

Brain region	Size (mm^3^)	L/R	*x*	*y*	*z*	*t*(17)
Body normal vs. (body scramble + object normal + face normal)						
Lateral occipitotemporal cortex	18.688	R	50	−65	10	4.92^***^
	2488	L	−46	−73	9	4.29^***^
Intraparietal sulcus	1840	R	13	−78	36	4.53^***^
Body normal vs. body scramble						
Lateral occipitotemporal cortex	51,936	R	52	−64	8	5.08^***^
	34,456	L	−47	−70	4	5.11^***^
Fusiform gyrus	10,728	R	41	−41	−18	4.69^***^
	4096	L	−43	−45	−18	4.28^***^
Precentral gyrus	1768	R	40	3	24	4.38^***^
Entorhinal cortex	1152	R	27	1	−27	4.65^***^
Intraparietal sulcus	2216	R	23	−75	36	4.33^***^

L/R indicates brain hemisphere. Coordinates are in MNI space and identify the peak voxel. *t*-values represent the average statistical value of the cluster.

^***^ *P* < 0.001.

#### Individual-subject analysis

The individual-subject analysis showed significant variability in brain responses to the body stimuli both across subjects and across runs within the same subject (see Supplementary Table S2). Using the strict contrast of normal bodies versus scrambled bodies, normal faces, and normal objects, the right EBA was localized in 14 of 18 participants and the left EBA in 11 of 18. While cluster coordinates were generally consistent across runs within participants, cluster sizes varied both across runs and across subjects.

**Figure 7:**
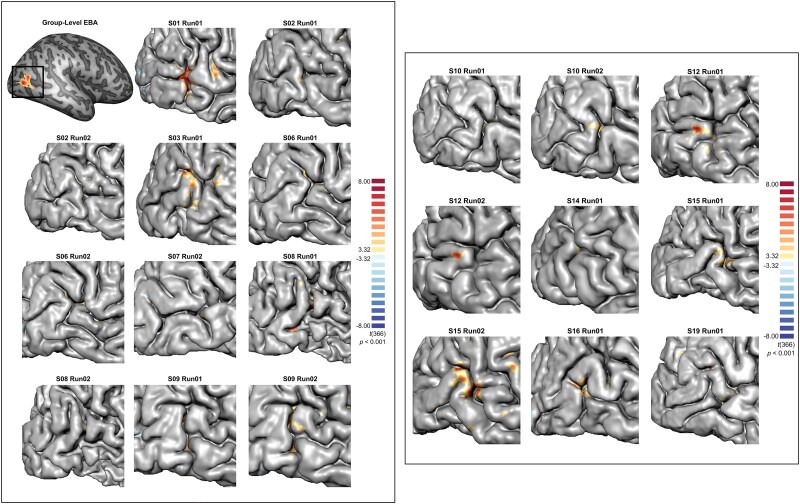
Anatomical localization of EBA. The anatomical location of the EBA in the right hemisphere of all included subjects and runs where the EBA cluster was visible on the cortex. S12 runs 1 and 2 are the only instances where the EBA cluster does not include the anterior occipital sulcus.

Despite variability in EBA coordinates across participants, projecting the statistical maps onto the cortical surface revealed significant consistency in anatomical location. All clusters, except for the right EBA in one subject, were located within the anterior occipital sulcus (AOS), which separates the occipital cortices from the temporal cortices (see Fig. 7).

## Discussion

The fMRI localizer experiment led to important results at the multisubject and individual subject level. The multisubject analysis identified the EBA with two different contrasts, a strict one comparing images of the human body against scrambled versions of those images, images of faces, and images of objects and a less strict contrast comparing human body images only against their scrambled counterparts. When comparing the results of the localizer against the coordinate review of Part II, several things are worth noting. The EBA location identified by the less strict contrast matched the results of the coordinate review more closely than the results of the strict contrast. This is consistent with the fact that the coordinate review included different coordinates identified with a large variety of contrasts. The results of the localizer also matched the in the finding from coordinate review that the left EBA is located more posteriorly than the right EBA. Finally, the EBA identified in the localizer experiment were more dorsally located than the results of the coordinate review. This again highlights the fact that the specific design of an EBA localizer can have a fundamental impact on the results, and this should be considered when localizing the EBA.

The individual-subject analysis results showed that the EBA could not be localized in all subjects and in some subjects not in both hemispheres, highlighting that there can also be various individual-level factors which can affect the results of an EBA localizer. Finally, where the region could be identified, the individual-subject analysis localized the EBA to the same anatomical region in almost all the participants.

## General discussion

Since its discovery in 2001 as a region of the brain selectively active for images of the human body, the extrastriate body area has been implicated in a wide range of cognitive processes and reported at varying anatomical locations. To date, however, no systematic review has comprehensively examined the different functions attributed to the EBA or the variability in its anatomical localization. The aim of this study was to provide an up-to-date, comprehensive overview of the literature on EBA function and localization, highlighting the region’s context-dependent roles. To this end, we conducted a literature review of neuroscientific articles reporting EBA activity, identifying 15 distinct research topics involving this region. Our results revealed substantial variability in both the stimuli and tasks used to localize the region, as well as considerable dispersion in reported coordinates Finally, we also conducted an fMRI study to investigate the location and extent of the EBA using a strict and highly controlled paradigm and analysis pipeline. This showed high inter- and intra-participant variability in EBA localization and extent, while simultaneously localizing the EBA to the anterior occipital sulcus.

### The role of the EBA

The results of the literature review show that our understanding of the function of the EBA has expanded greatly since then. The region has been found to show greater activity for videos of bodies compared to static images (Pitcher et al. 2019) and to be selective for non-visual stimuli representing bodies, such as haptic body representation (Constantini et al. 2011). There is a large body of contrasting results about whether the EBA differentiates between the self and others, with some arguing that this distinction is processed by a region near but separate from the EBA (Hodzic et al. 2008, Ogawa and Matsuyama 2023). Primarily the left EBA and its connectivity to the parietal cortex seem to be involved in body representation and ownership (Limanowski and Blankenburg 2015; Limanowski and Blankenburg 2017; Moayedi et al. 2021). Additionally, the EBA generally shows greater activity for emotional body expressions than neutral ones (Van den Stock et al. 2012; Ren et al. 2022) and is also involved in the related social interaction perception (Landsiedel et al. 2022; Gandolfo et al. 2024). Furthermore, by providing information about body kinematics and postures, the EBA is a key node in action perception (Zimmermann et al. 2012; Ma et al. 2018) and has itself been found to differentiate between action goals and semantics (Herrington et al. 2012, Kubiak Kroliczak et al. 2016). Finally, the region has been found to be involved in a variety of clinical disorders (eg Suchan et al. 2010; van Nuenen et al. 2012a), as well as in aesthetic decisions about bodies (Cazzato et al. 2014; Cazzato et al. 2016; Cazzato et al. 2017).

It should thus be clear that the EBA is not simply a categorical visual representation area, but rather that it is a highly interconnected region with functional properties dependent on a given task and context (de Lange and Bekkering 2011; Li et al. 2023). The results of the literature review clearly align with a move toward a behavior-driven understanding of categorical representation in the brain (Bracci & Op de Beeck, et al. 2022; Li et al. 2025; Ritchie et al. 2025). The function associated with the EBA remain tied to the concept of bodies, but it is unclear how that function is implemented, what features matter for body sensitivity, what computations take place, and what if anything makes those features and computations presumably body specific. For example, one recent proposal directly addressing EBA selectivity for dynamic images is that EBA computes biomechanical properties of the human body (Marrazzo et al. 2025).

### Location of the EBA

Concerning the localization of the EBA, the coordinate review and the fMRI experiment agreed on several points. The results showed that generally, the right EBA covers a larger volume than the left EBA and is more anterior to the left. The coordinate review and fMRI experiment also broadly aligned in the average coordinates reported for the EBA. However, there was quite a significant difference between the two contrasts used to define the EBA in the present fMRI experiment. If the EBA was defined by comparing videos of bodies with matching pixel-scrambled videos, this resulted in much larger clusters of significant activity than if the region was defined by contrasting body videos with the scrambled videos along with videos of faces and videos of objects. The clusters resulting from the second contrast were much smaller, particularly in the left hemisphere. Moreover, the left hemisphere cluster was also more dorsally located.

This difference in results between the two explored contrasts can help explain the wide range of average and peak coordinates identified by the coordinate review as well as why the meta-analyses find the EBA to span such a wide range. Already a relatively minor change in our analysis pipeline resulted in significantly different results. Our review shows that a host of different stimuli have been used to identify regions in the brain, which have been labeled as the EBA. One can thus expect that a region identified using a contrast of static images of bodies and chairs (the most common localizer stimuli) will differ significantly from one localized using, for example, words associated with actions away or toward the body (Rueschemeyer et al. 2010). Additionally, in many studies the EBA label is also used for regions in the absence of any functional localizers and is instead applied based only on identified location and purported function.

The present fMRI data collection also led to several other important findings. Firstly, we were unable to localize the EBA among all our participants (the EBA was found in 14/18 participants in at least one hemisphere). Failure to identify the EBA in some participants could be due to several reasons, including participant attention, inherent characteristics, or external variables such as participant caffeine intake—all of which warrant further, systematic exploration. Secondly, we saw differences in EBA size not only between participants but also between individual runs of single participants. This again suggests that EBA activity may not only be affected by individual characteristics (see also Aleong and Paus 2010; Willems et al. 2010) but also by contextual factors, such as attention. Finally, for all participants for whom we were able to identify the EBA except one, the area was located bilaterally in the same anatomical location—within the anterior occipital sulcus. This finding may be particularly useful to the field of translational neuroscience and to investigations into the patterns of body-sensitive areas across species, for instance comparing this to the known body-selective patches in the macaque temporal cortex (Vogels 2022). Concerning this last finding, it may be more appropriate to say that we identified a region in the participants’ anterior occipital sulci, which showed significantly higher activity for videos of bodies compared to videos of objects, faces, and scrambled bodies. The results of our review do not guarantee that had we localized the region with a different set of stimuli or included a different task than passive attention, we would have found significant clusters in the AOS.

## Conclusion

Past research has been using the term “EBA” using widely different stimuli, localized to non-overlapping regions of the cortex with disparate functions. Here, we presented an overview of the functional properties of the extrastriate body area as well as its various reported locations throughout the cortex. It can hopefully serve as a guide for future investigations of the region, whether to help localize the EBA for neurostimulation studies or to highlight relevant gaps or inconsistencies in the literature. Finally, our review suggests that localization issues may be best be approached in the context of studies focused on understanding the role of EBA in behavior.

## Supplementary Material

Smekal_CerCor_SupplementaryMaterials_bhag078
