# Supplementary material for: From localization to distribution: revisiting the functional organization of the extrastriate body area
Source: Cereb Cortex. 2026 Jul 9;36(6):bhag078. doi: 10.1093/cercor/bhag078 (PMC13347403; doi:10.1093/cercor/bhag078)
Supplement: Smekal_CerCor_SupplementaryMaterials_bhag078 [file smekal_cercor_supplementarymaterials_bhag078.pdf]

Supplementary Materials

From localization to distribution: Revisiting the functional organization of the Extrastriate Body Area

Vojtěch Smekal[1], Lena L. M. Beckers[1], Marta Poyo Solanas[1] & Beatrice de Gelder[1]*
[1] Cognitive Neuroscience Department, Maastricht University, Maastricht 6229EV, the Netherlands

*Corresponding author: b.degelder@maastrichtuniversity.nl

Topic	Number of Coordinate Sets	Articles
Action processing (31)	64	Abreu et al. (2012), Adamovich et al. (2009), Amoruso et al. (2011), Arzy et al. (2006), Baldassano et al. (2016), Calmels et al. (2014), Candidi et al. (2008), Cheng et al. (2007), de Vega et al. (2014), Di Nota et al. (2016), Gu et al. (2021), Hamilton et al. (2006), Herrington et al. (2012), Kable and Chatterjee (2006), Kubiak and Króliczak (2016), Kühn et al. (2011), Ma et al. (2018), Marsh et al. (2010), Okamoto et al. (2021), Piefke et al. (2009), Romaiguère et al. (2014), Sasaki et al. (2018), Schubotz and von Cramon (2004), Simos et al. (2017), Takahashi et al. (2008), Urgesi et al. (2006), van Elk (2014), van Steenbergen et al. (2017), Wiggett and Downing (2011), Zimmermann et al. (2012, 2016)
Body ownership (19)	42	Araujo et al. (2015), Bao et al. (2021), Beck et al. (2015), David et al. (2007), Di Vita et al. (2016), Golaszewski et al. (2013), Hamamoto et al. (2023), Hummel et al. (2013), Kontaris et al. (2009), Limanowski and Blankenburg (2015, 2016, 2017), Limanowski et al. (2014), Moayedi et al. (2021), Seghezzi et al. (2019), Sonobe et al. (2023), Tomasino et al. (2012), Wold et al. (2014), Yomogida et al. (2010)
Body selectivity (58)	204	Aleong and Paus (2010), Andres et al. (2012), Astafiev et al. (2004), Bauser and Suchan (2015), Blanke et al. (2010), Bracci et al. (2010), Brandman and Yovel (2016), Caspari et al. (2014), Constantini et al. (2011), Corradi-Dell'Acqua et al. (2015), Cziraki et al. (2010), Downing and Peelen (2016), Downing et al. (2007), Downing et al. (2001, 2006), Duarte et al. (2022), Ewbank et al. (2011), Felician et al. (2009), Foster et al. (2019, 2022), Fusco et al. (2022), Gandolfo and Downing (2019), Gilaie-Dotan et al. (2015), Heyda et al. (2010), Kemmerer and Tranel (2008), Kim et al. (2020), Kitada et al. (2009), Labek et al. (2017), Lacey et al. (2017), Li et al. (2023), Marrazzo et al. (2023), Michels et al. (2005), Moro et al. (2008), Morris et al. (2006), Murty et al. (2021), Peelen and Downing (2005), Perruchoud et al. (2016),

		Pitcher et al. (2009, 2012), Pitcher et al. (2019), Pourtois et al. (2007), Poyo Solanas et al. (2020a), Rueschemeyer et al. (2010), Saygin et al. (2011), Spiridon et al. (2006), Stigliani et al. (2015), Taylor and Downing (2011), Taylor et al. (2007, 2009), Thompson and Baccus (2012), Urgesi et al. (2004, 2007), van Koningsbruggen et al. (2013), Van den Stock, Tamietto, et al. (2014), Vangeneugden et al. (2014), Willems et al. (2010), Zeharia et al. (2019)
Clinical (28)	159	Brown et al. (2019), Canário et al. (2023), Castellini et al. (2013), Esposito et al. (2018), Grossi et al. (2017), Haipt et al. (2022), Helmich et al. (2007), Kikuchi et al. (2017), Kodama et al. (2018), Lavagnino et al. (2014), Mattavelli et al. (2019), Okamoto et al. (2014, 2017, 2018), Paul et al. (2019), Press et al. (2022, 2023), Suchan et al. (2010, 2013), Takahashi et al. (2010, 2012), van Nuenen, Helmich, Buenen, et al. (2012), van Nuenen, Helmich, Ferraye, et al. (2012), Van den Stock, van de Riet, et al. (2008), van der Stouwe et al. (2018), Vocks, Busch, Grönermeyer, et al. (2010), Vocks, Busch, Schulte, et al. (2010), Vocks et al. (2011)
Connectivity (2)	5	Rebollo et al. (2018), Zimmermann et al. (2018)
Development (8)	31	Kitada et al. (2014), Kosakowski et al. (2022), Peelen et al. (2009), Pelphrey et al. (2009), Ross et al. (2014), Striem-Amit and Amedi (2014), Walbrin et al. (2020), Yizhar et al. (2023)
EEG (16)	1	Caggiano et al. (2022), Costa et al. (2023), Espírito Santo, Chen, and Schürmann (2017), Espírito Santo, Maxim, and Schürmann (2017), Giabbiconi et al. (2016), Mado Proverbio et al. (2008, 2012), Möhring et al. (2014), Moreau, Candidi, et al. (2020), Moreau, Parrotta, et al. (2020), Moreau et al. (2017), Ortigue and Bianchi-Demicheli (2008), Quinzi et al. (2019), Sadeh et al. (2011), Salgues et al. (2021), Taylor et al. (2010)
Emotion perception (19)	86	Caillaud et al. (2020), Engelen et al. (2015), Goldberg et al. (2015), Jung et al. (2020), Kret, Denollet, et al. (2011), Kret, Pichon, et al.

		(2011a, 2011b), Lamm and Decety (2008), Peelen et al. (2007), Ren et al. (2022), Ross et al. (2019), Sinke et al. (2012), Vachon-Presseau et al. (2012), Vaessen et al. (2023), van de Riet et al. (2009), Van de Vliet et al. (2018), Van den Stock et al. (2012, 2013), Yang et al. (2018)
fNIRS (3)	11	Ishizu et al. (2009), Schneider et al. (2014), Shimada (2013)
MEG (5)	2	Buchholz et al. (2019), Ishizu (2013), Ishizu et al. (2010), Meeren et al. (2013), Nakamura et al. (2015)
Multisensory processing (4)	54	Atilgan et al. (2023), Beer et al. (2013), Fourcade et al. (2022), Kim et al. (2019)
Neuroaesthetics (6)	3	Calvo-Merino et al. (2010), Cattaneo (2020), Cazzato et al. (2014, 2015, 2016), Holliday et al. (2011)
Self vs. Other (17)	66	Arora et al. (2017), Callan et al. (2012), Carey et al. (2019), Cazzato et al. (2015), Chan et al. (2004), David et al. (2009), De Bellis et al. (2017), Dumontheil et al. (2010), Hodzic et al. (2008, 2009), Jackson et al. (2006), Myers and Sowden (2008), Ogawa and Matsuyama (2023), Pann et al. (2021), Saxe et al. (2006), Schurz et al. (2013), Vocks, Busch, Grönermeyer, et al. (2010)
Social interaction perception (12)	40	Bellot et al. (2021), Gandolfo et al. (2024), Greven and Ramsey (2017), Hahn and O'Toole (2017), Landsiedel et al. (2022), McMahon et al. (2023), Okamoto et al. (2020), Quadflieg et al. (2015), Saxe (2006), Sinke et al. (2010), Van den Stock et al. (2015), Walbrin and Koldewyn (2019)
Subdivisions of EBA (2)	0	Ferri et al. (2013), Weiner and Grill-Spector (2011)

Table S1. Literature review by topic. The articles identified in the literature sorted by theme (first column). The numbers in brackets in the first column indicate the number of papers included in that topic. The second column indicates the number of EBA coordinates (individual or group) extracted from the papers within that topic. The papers within the third column are sorted alphabetically for each topic.

Subject	Run	L/R	x	y	z	Size (mm^3)	Anatomical location
S01	01	R	41	-68	5	42,352	Junction of LOS and AOS-i
		L	-36	-75	-2	31,128	Junction of LOS and AOS-i
S02	02	R	38	-68	17	992	AOS
S03	01	R	53	-66	22	7,576	Rostral MOG and dorsal AOS
		L	-51	-72	4	4,288	Junction of LOS, AOS, AOS-i
S06	01	R	43	-58	16	2,200	Dorsal AOS
			43	-44	7	1,184	
			47	-67	7	1,592	
		L	-47	-56	13	3,208	Junction of ITS and AOS; banks of IOG and ITG
			-56	-66	14	4,480	
	02	R	39	-61	8	9,000	Junction of AOS and STS
			46	-45	6	2,672	
		L	-47	-57	16	3,000	Junction of ITS and AOS; banks of IOG and ITG
			-45	-75	-1	1,208	
			-62	-57	21	2,960	
S07	02	R	49	-69	-1	2,944	AOS-i
S08	01	R	50	-60	9	5,152	AOS and AOS-i
			41	-71	-5	3,176	
		L	-41	-71	8	1,032	Junction of AOS and LOS
	02	R	49	-61	8	1,232	AOS
S09	01	R	46	-65	9	1,896	AOS
			41	-60	-5	1,968	
		L	-48	-72	7	1,592	AOS
	02	R	46	-64	8	9,840	Junction of AOS and ITS
			40	-59	-4	5,304	
		L	-51	-73	9	3,296	AOS-I and AOS
			-46	-63	17	1,096	
			-56	-65	15	1,344	
S10	01	R	44	-61	1	5,744	Junction of AOS and STS-h
		L	-52	-60	7	1,792	AOS
	02	R	45	-62	1	10,240	Junction of AOS and STS-h
		L	-53	-67	5	3,816	AOS
S12	01	R	50	-72	12	4,648	MOG
		L	-43	-74	9	1,040	Dorsal, caudal AOS-s
	02	R	51	-71	12	1,640	MOG
		L	-43	-74	9	1,672	Dorsal, caudal, AOS-s
S13	01	L	-40	-64	18	1,280	AOS
			-48	-70	17	1,272	
	02	R	48	-58	19	856	
S14	01	R	57	-69	-2	1,592	AOS
S15	01	R	50	-60	16	12,328	AOS and ITS-p
		L	-53	-66	13	7,784	AOS
	02	R	51	-61	16	24,320	AOS and ITS-p
		L	-52	-67	13	3,848	AOS
			-59	-65	3	5,640	

S16	01	R	45	-74	8	11,064	Junction of AOS, LOS, and ITS
		L	-57	-67	7	10,776	
S19	01	R	48	-64	17	6,016	AOS, STS, and TOTZ
		L	-49	-60	8	7,696	Junction of AOS and STS
	02	R	52	-67	19	2,264	AOS and STS
		L	-48	-60	8	944	Junction of AOS and STS

Table S2. Anatomical locations of EBA in all subjects. Average coordinates of clusters showing a significant *p*-value for the comparison of normal body > normal object + normal face + scrambled body for each run of each subject in the localizer experiment, where a significant cluster was identified. Coordinates are in MNI space. Abbreviations: AOS – anterior occipital sulcus; AOS-i – anterior occipital sulcus, inferior ramus; AOS-s – anterior occipital sulcus, superior ramus; IOG – inferior occipital gyrus; ITG – inferior temporal gyrus; ITS – inferior temporal sulcus; ITS-p – inferior temporal sulcus, posterior segment; LOS – lateral occipital sulcus; MOG – middle occipital gyrus; STS – superior temporal sulcus; STS-h – superior temporal sulcus, horizontal segment; TOTZ – temporoccipital transition zone.

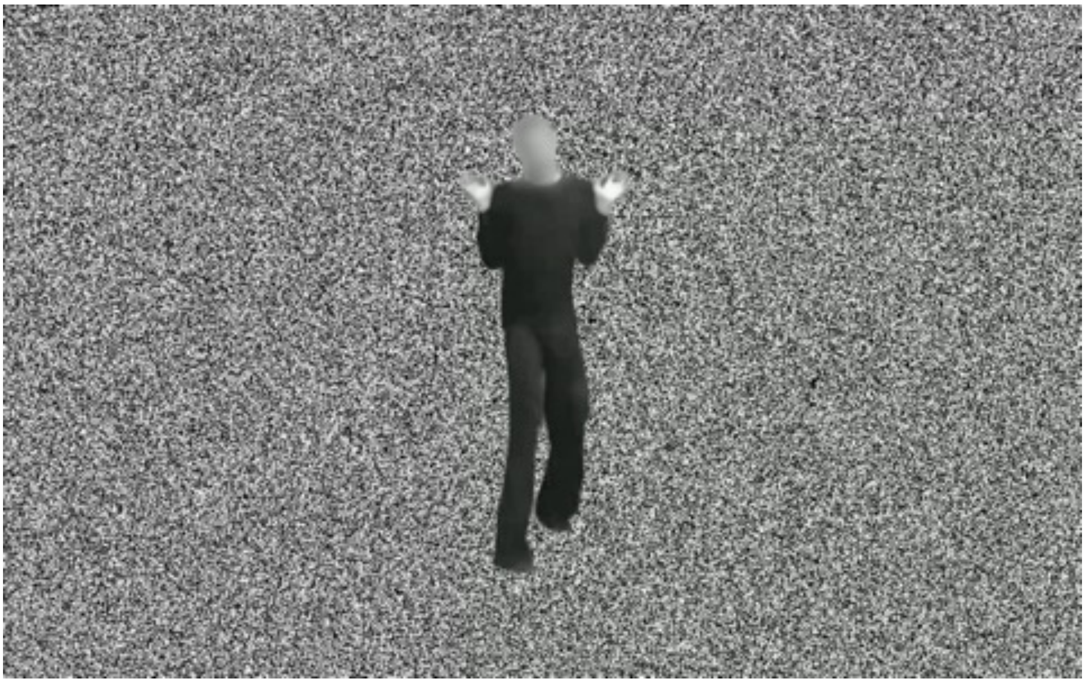

Figure S1. Example body stimulus. An example frame from the dynamic body stimuli used in the EBA localizer in Part III. The background consisted of a dynamic noise background and the face was blurred throughout the video.

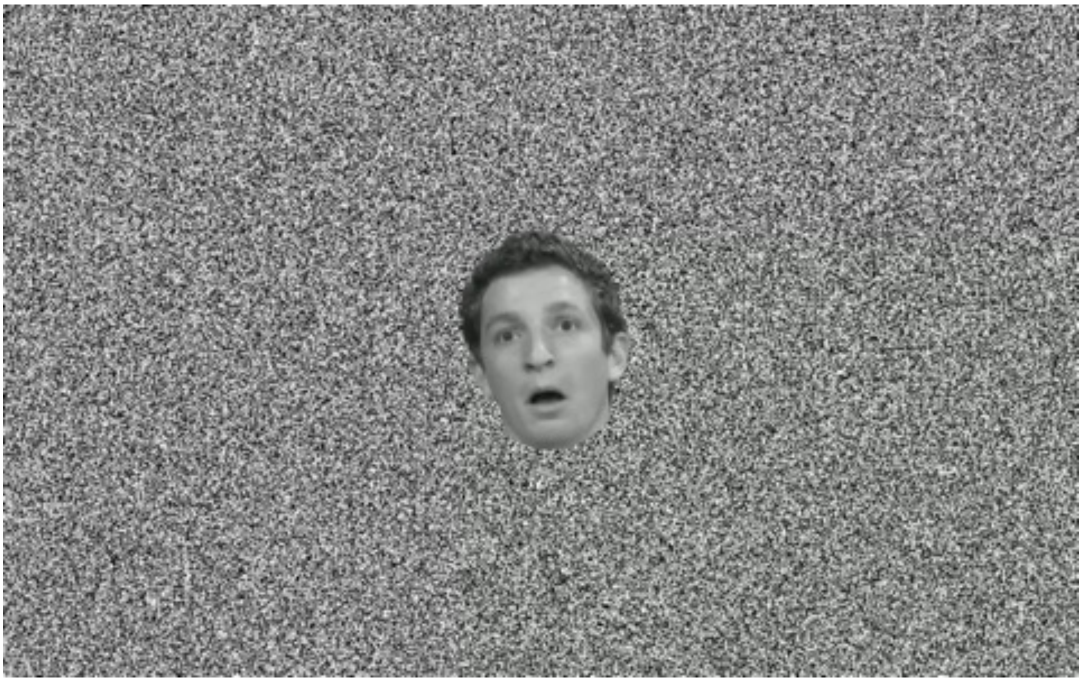

Figure S2. Example face stimulus. An example frame from the dynamic face stimuli used in the EBA localizer in Part III. The background consisted of a dynamic noise background.

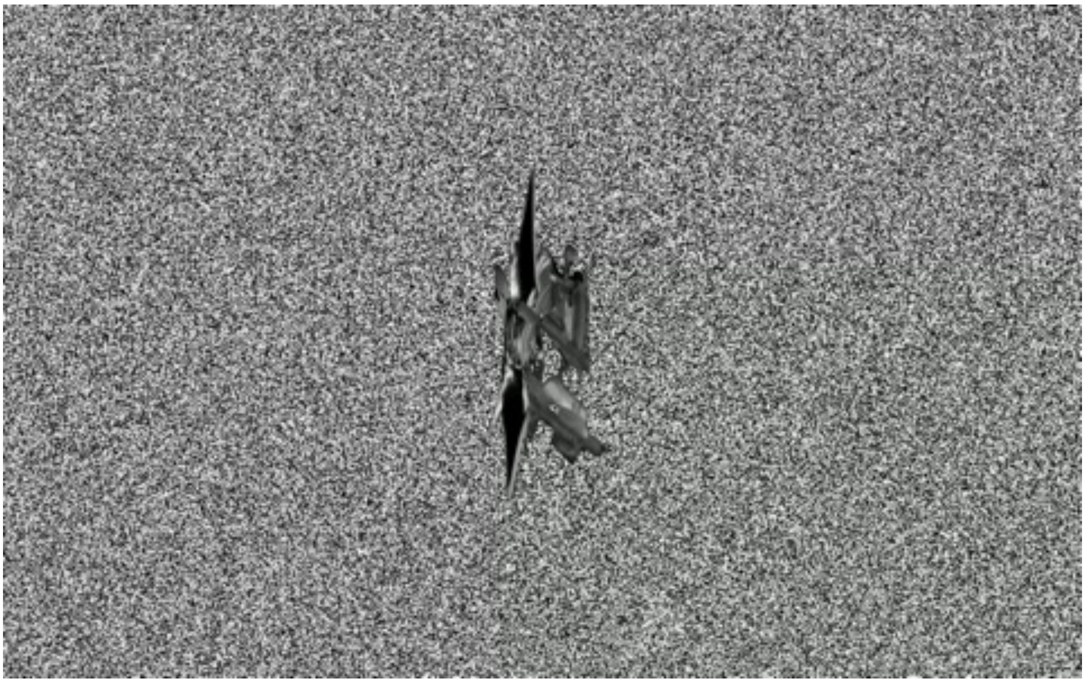

Figure S3. Example object stimulus. An example frame from the dynamic object stimuli used in the EBA localizer in Part III. The object was designed to have no clear purpose. The background consisted of a dynamic noise background.

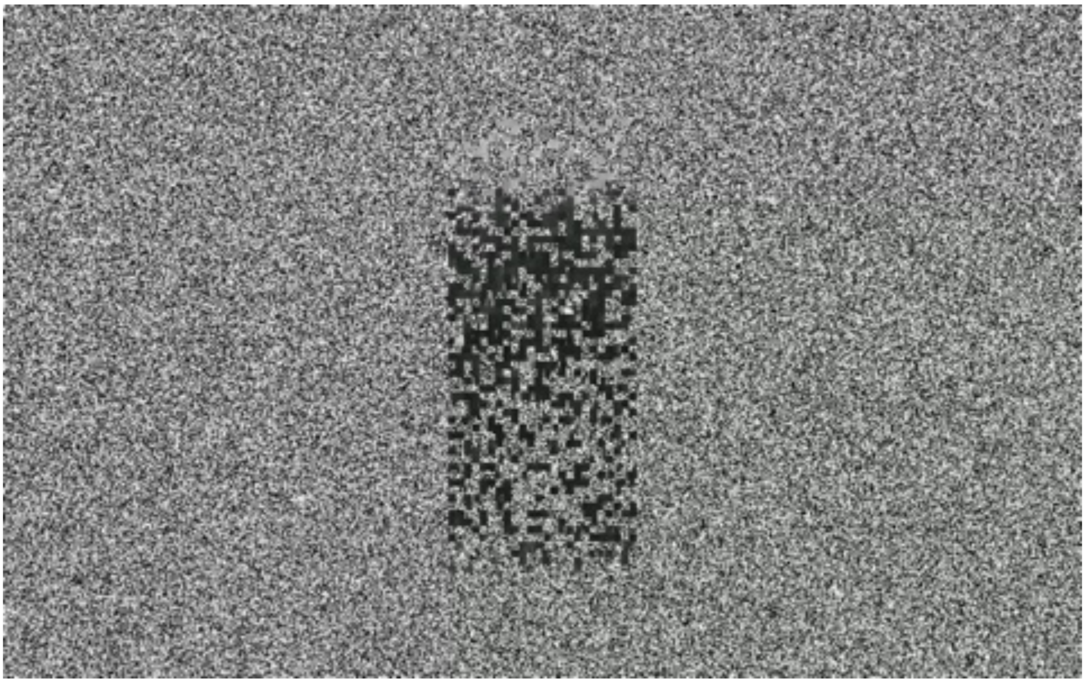

Figure S4. Example scrambled body stimulus. An example frame from the dynamic scrambled body stimuli used in the EBA localizer in Part III. The pixels composing a full body with face blurred were shifted in location, while maintaining the same visual angle dimensions.
